# A Robust and Accurate Method for Feature Selection and Prioritization from Multi-Class OMICs Data

**DOI:** 10.1371/journal.pone.0107801

**Published:** 2014-09-23

**Authors:** Vittorio Fortino, Pia Kinaret, Nanna Fyhrquist, Harri Alenius, Dario Greco

**Affiliations:** 1 Unit of Systems Toxicology, Finnish Institute of Occupational Health (FIOH), Helsinki, Finland; 2 Nanosafety Centre, Finnish Institute of Occupational Health (FIOH), Helsinki, Finland; Children's Medical Research Institute, Australia

## Abstract

Selecting relevant features is a common task in most OMICs data analysis, where the aim is to identify a small set of key features to be used as biomarkers. To this end, two alternative but equally valid methods are mainly available, namely the univariate (filter) or the multivariate (wrapper) approach. The stability of the selected lists of features is an often neglected but very important requirement. If the same features are selected in multiple independent iterations, they more likely are reliable biomarkers. In this study, we developed and evaluated the performance of a novel method for feature selection and prioritization, aiming at generating robust and stable sets of features with high predictive power. The proposed method uses the fuzzy logic for a first unbiased feature selection and a Random Forest built from conditional inference trees to prioritize the candidate discriminant features. Analyzing several multi-class gene expression microarray data sets, we demonstrate that our technique provides equal or better classification performance and a greater stability as compared to other Random Forest-based feature selection methods.

## Introduction

Identifying discriminant features, for instance from transcriptomics experiments, and modelling classifiers based on them are fundamental tasks when the aim is to highlight biomarkers (*e.g.* genes or transcripts discriminating healthy from diseased samples). Indeed, clinical classification based on high throughput molecular profiling has been already explored for a number of complex diseases, such as cancer [1], [2]. These studies become crucial also in terms of public health when such approaches are considered for clinical practice [3]. On the other hand, concerns about the stability and reproducibility of microarray results have been raised, despite the huge propagation of the gene selection methods in biomarker discovery [4] and interest on this topic seems to be increasing [5], [6]. The most frequently used feature selection techniques include univariate (filter), and multivariate (wrapper), approaches. Univariate techniques, such as the formal statistical hypothesis testing or, more in general, the ranking methods, test each feature separately. Multivariate techniques assess the relevance of groups of features simultaneously, by using selection methods (*e.g.* forward or backward selection) coupled with machine learning techniques such as logistic regression, support vector machines (SVM) or random forests (RF) [7]–[9]. Unfortunately, multivariate methods tend to identify different subsets of candidate biomarkers with equal accuracy, even when feature selection algorithms are used on the same data [5], [6]. This is particularly true for feature selection problems in OMICs data analysis, where the number of investigated features is much larger than the number of samples. Multiple stability issues can in fact affect these data sets, and the data sets can contain large number of redundant features [10].

The aim of this study was to develop a feature selection and prioritization framework capable of guaranteeing high stability as well as high classification performance. First, an unsupervised fuzzy pattern discovery method [11] is used to discretize the gene expression data and to identify fuzzy-based feature signatures called fuzzy patterns (FP). Each FP summarizes the most relevant features of each class. Next, a Random Forest (RF) procedure, where the prior knowledge of the FPs is used to enhance the performance of each tree within the classifier, is run on the original data. Last, a permutation based variable importance measure is used to rank the selected features and produce the final prioritized feature list.

We tested our fuzzy pattern – random forest (FPRF) procedure, implemented in R language [12], as well as two widely used methods for feature selection based on RFs and also implemented in R - varSelRF [8] and Boruta [9], on several gene expression microarray data sets investigating human samples in different pathophysiological conditions. The basic idea of varSelRF is to execute a backward iterative selection process that exploits the measures of variable importance, computed by RF based on CART trees [13], whereas Boruta uses an iteration process that removes, at each run, the features with less contribution to classification accuracy by introducing random variables for the competition [10].

## Materials and Methods

### The FPRF algorithm

**Figure 1:**
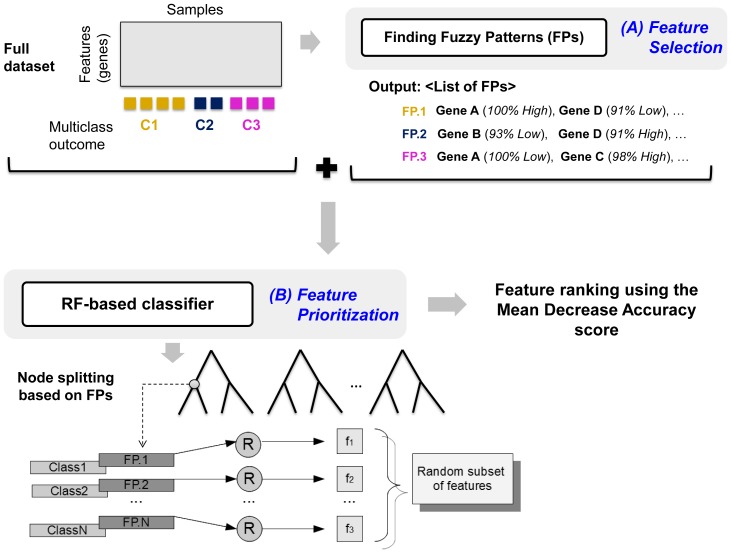
Feature selection and prioritization schema. The feature selection step is based on a fuzzy pattern discovery method implemented into the R-package *DFP*. This method is able to identify the most relevant class-specific features, forming a fuzzy pattern, for each class (A). The selected features are then ranked using a modified RF procedure that exploits the fuzzy patterns to improve the performance of the decision trees grown from large subset of samples and features (B). The RF algorithm works on the gene expression dataset given by the union of all features selected in the first step. Furthermore, the knowledge of the fuzzy-based feature signatures is exploited in order to have a different random selection procedure. For each node, the subset of features used for the splitting process is composed by a random selected feature from each fuzzy feature pattern.

Figure 1 depicts the proposed schema of feature selection and prioritization, when dealing with multiclass high-throughput data. The first step consists of a fuzzy pattern discovery method, implemented in the R package *DFP* [11], which is used to select large subsets of relevant and independent class-specific features indicated as FPs (see Figure S1 for details). First, the membership function for each feature is computed and each value is consequently transformed into a linguistic label (*i.e.* “Low”, “Medium”, “High” and their intersections “Low-Medium” and “Medium-High”). The output is a discretized (fuzzyfied) dataset that is only used to generate the FPs. A FP is a large set of features whose fuzzyfied pattern is correlated with a specific class (or a biological state of interest). The union of all FPs forms the set of selected features (step 1 in Figure 1). At the second step, the selected features are prioritized using a RF-based classifier. This is achieved by using a modified RF algorithm that helps reducing the risk of considering redundant features for the node splitting process, improving the accuracy of the decision trees and the final rank of the features.

### The discretization and selection steps

As shown in Figure S1, the full dataset is discretized into fuzzy labels through the application of membership functions (MFs). A MF is a curve that defines, for each feature in the data set, how each numerical value in the input space is mapped to a membership value or linguistic label (*i.e.*, “Low”, “Medium” and “High”). In the *DFP* package, two types of membership functions are used: i) the polynomial approximation of a Gaussian membership function to model the range of ‘normal’ expression levels of a gene and ii) the polynomial approximation of two sigmoidal membership functions, which are able to specify asymmetric membership functions for the ‘low’ and ‘high’ expression levels. After the discretization step, the FP for each class (or outcome) is computed by selecting the genes with highly frequent discretized label in at least one class (Figure S1). The discretization and selection step is based on two parameters (*zeta*, *piVal*). The *zeta* parameter is a threshold used in the membership functions to label the float values with a discrete value, and is thus important for the fuzzy discretization process. The parameter *piVal*, on the other hand, specifies the percentage of values of a class to determine the FPs. Essentially, these two parameters influence the number of features included in each FP. Smaller values of *zeta* and bigger values of *piVal* result in smaller FPs containing less features. While too small FPs can resolve in some empty FPs, bigger FPs might include less relevant features. In our experiments, we have preferred to work with bigger FPs by using the parameter configurations shown in Table S1. Since in FPRF the predictive power of the features is evaluated in the RF-based classifier, the less informative features will rank low in the final list. Nevertheless, nominally less predictive features that become important in combination with others will have a higher rank in the output.

### The RF-based feature ranking method

We used a RF-based classifier to rank the features selected by the fuzzy pattern discovery method. The RF implementation utilized is based on unbiased classification trees, as implemented in the ctree function in the R package *party* [14], [15]. The feature importance is usually evaluated through methods such as the Gini importance and the “mean decrease in accuracy” or “permutation” test, available in the package *randomForest* [16]. Similarly, a permutation importance measure for cforest is available in *party*. Since, the Gini importance criterion may lead to biased results [17], [18], we used the permutation accuracy importance score to evaluate the selected features.

Furthermore, the package *party* was modified in order to introduce a new RF procedure that exploits the information of FPs to improve the accuracy of the decision trees. At each node, the standard RF procedure, selects M variables at random and searches for the best split over them. This procedure is referred to as node splitting process. The new RF procedure simply replaces the random selection of M features with a new process that picks one random relevant feature from each fuzzy pattern. The basic idea is to increase the number of relevant features selected for the node splitting process, restricting the random feature selection from the FPs. Indeed, we observed by internal studies that the RF models built with the proposed RF procedure are significantly better than those obtained with the standard procedure. In a more detail, the random subset of features at each node splitting is built as follows:
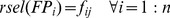
(1)


Where 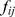 is the feature randomly selected from the 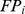 and the index 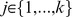. *k* is the number of genes in 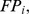 and *n* is the number of FPs ( =  #number of classes). The number of trees used by the RF classifiers to rank the selected features is a relevant parameter, and we used 1000 trees for all the data sets. This value was also used to evaluate the accuracy of all trained RF-based classifiers. The methods varSelRF and Boruta were used with default parameters.

### Datasets analyzed

Four multi-class gene expression microarray data sets were analyzed to evaluate the performances of FPRF. The first data set is a compendium of human peripheral blood mononuclear cells (PBMC) samples, consisting of seven classes, which were generated by integrating multiple independent publicly available series (Table S2) from the NCBI GEO repository (http://www.ncbi.nlm.nih.gov/geo/). The second data set is a series of transcriptomics profiles of bone marrow cells (BM) from patients with different subtypes of acute lymphoblastic leukemia (ALL) [19] (http://www.stjuderesearch.org/site/data/ALL1). In the original study, the dataset Leukemia has been divided into six diagnostic groups (BCR-ABL, E2A-PBX1, Hyperdiploid>50, MLL, T-ALL and TELL-AML), and one that contains samples that did not fit into any one of the above groups. But, in our study we preferred to consider only those samples that were belonging to one of 6 categories (276). In addition, two data sets from the IMPROVER challenge [20], profiling respectively lung cancer (four classes,, GSE43580) and psoriasis specimens (three classes, GSE13355 and GSE14905), respectively, were also analyzed. The preprocessing of all the data sets has followed a similar procedure. First, the raw data (.CEL files) were imported into R v. 3.0.0 and their quality were checked with the packages *AffyQCReport* (http://www.bioconductor.org/packages/2.12/bioc/html/affyQCReport.html) and *affyPLM* (http://www.bioconductor.org/packages/2.12/bioc/html/affyPLM.html). Subsequently, the background correction, normalization and summarization of the gene expression values were performed according to the RMA algorithm implemented in the package *affy* [21]. Alternative CDFs v.18 (http://brainarray.mbni.med.umich.edu/brainarray/default.asp) were used for probe annotations based on the Ensembl Gene (http://www.ensembl.org/index.html) or the Entrez Gene (http://www.ncbi.nlm.nih.gov/gene) databases. For the PBMC compendium, the technical biases associated with the series and the platform (different Affymetrix chipsets) were corrected using the ComBat algorithm [22] implemented in the R *sva* package [23], [24]. Finally, the genes with low variability across the samples were eliminated.

### Testing and assessment of the results

**Figure 2:**
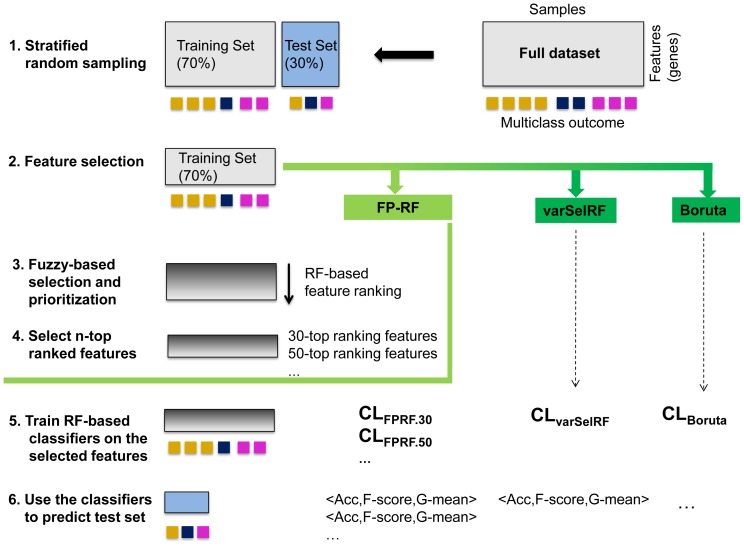
Evaluation process. The full dataset is a matrix with thousands of features (*e.g.* genes) in rows and tens or hundreds of samples (belonging to different classes) in columns. For each sample, the outcome (class) is given. The dataset is randomly divided into training and test sets using a stratified random selection (1). Within the training set, relevant features are selected using the compared methods (2). The FPRF method identifies a wide set of relevant features using a fuzzy pattern discovery technique and ranks them applying a RF-based procedure (3). The most n-relevant features are then selected with n = 30, 50, 100, 150 and 200 (4). The different sets of features are used to evaluate the stability and the corresponding classification performance. For each set of selected features an RF-based classifier is trained on the training set (5). After training, the classifiers are asked to predict the outcome of the test set patients (6). The predicted outcome is compared with the true outcome and the number of correctly classified samples is noted. Steps 1–6 are repeated 30 times, and the resulting evaluation metrics are obtained by averaging over the 30 runs.

Figure 2 depicts the general schema used to evaluate and compare our method with varSelRF and Boruta. After having randomly selected and left aside the 30% of the data, the remaining 70% of the data was used to generate the sets of selected, relevant features (or genes). Since our method runs feature selection and prioritization, the generated ranked lists were cut-off at different points (or lengths) in order to produce lists of features of different sizes, referred to as n-top ranked feature lists. Next, the classification accuracy of each n-top ranked feature list and the feature lists obtained with varSelRF and Boruta were evaluated. For each list of features, a RF classifier was first trained on the randomly selected 70% of the data set, and then tested on the remaining 30% of the data, in order to get the post-selection classification accuracy. This process was repeated 30 times in order to assess the resulting classification metrics and the stability of the selected features.

The execution time for each run was recorded. All the analyses were performed with a quad-core Intel Core i7 3.4 GHz and 32GB DDR3 RAM running Apple Mac OS X v.10.9.2 and R 3.0.2.

### Evaluation Metrics

The evaluation metrics used in this study aimed at assessing the classification accuracy and the stability of the compared methods. The stability was evaluated by comparing the lists of features selected by each method over 30 bootstrap iterations. From these, we looked for significantly self-consistent features [10], that were selected more frequently by bootstrap iterations than what would be expected at random. For each method, we computed the ratio between the number of self-consistent features and the total number of features selected over the 30 bootstrap iterations.

The post-classification accuracy was estimated by the F-score and the G-mean metrics, which are particularly appropriate when unbalance multi-class problems are considered [25]. The F-score is based on the F-measure, which is calculated as follows: 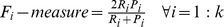
(2)


Where 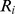 and 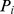 are the recall and the precision, respectively, for the *i^th^* class, and *k* is the number of class labels. A high F-measure value guarantees that both recall and precision are reasonably high. The extended F-measure metric for the multi-class case is described as follows: 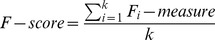
(3)


The G-mean function is instead defined as the geometric mean of the recalls across all the classes.
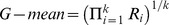
(4)


In addition to these two main metrics, we also report the overall accuracy.

## Results and Discussion

We compared the novel feature selection and prioritization method FPRF with two common RF-based feature selection methods, varSelRF and Boruta. We used publicly available gene expression datasets, and evaluated the classification performance and the stability of the selected features following the schema illustrated in Figure 2.

### Empirical evaluation - accuracy

A common method for the assessment and tuning of feature selection methods consists in evaluating the accuracy/error of a classifier that is trained using only the selected features. At each bootstrap iteration, the features that were selected from the training set were used to model a RF-based classifier on the test set. The test set contained samples that were not present in the corresponding training set, and therefore not considered during the feature selection step. Since our method selects and ranks the features, we trained different RF-based classifiers on the training set, selecting different cut points: the first 2, 3, 4, 5, 10, 20, 30, 50, 150, 200 and 250 top-ranked features. We then compared the classification performance of the classifiers FPRF.2–250, with those obtained using the features selected from varSelRF and Boruta. The idea was to compare our method with Boruta and varSelRF, and simultaneously find the best cut points for each analyzed datasets. To impartially assess the classification performance, we used three extended measures, namely, Acc, G-mean (*G*) and F-score (*F*), as defined in the Material and Methods.

Table 1 reports the results of varSelRF and Boruta, along with the different n-top ranked feature lists generated by FPRF on the four analyzed data sets. The evaluation metrics were averaged over 30 bootstraps. The RF-based classifiers that were trained with less than or equal to 50-top ranked feature lists exhibited the best classification performance among the different n-top ranked feature lists (Table 2). In the Lekumia dataset the Accuracy, F-score and G-mean values compiled for FPRF.10 and FPRF.20 are almost always bigger than the same values compiled for FPRF.3–5 and FPRF.30–250. Of note, the Leukemia dataset was sensitive to class imbalance, while other datasets were not, as shown by the difference between the Accuracy as well as the F-score and G-mean values. An Accuracy value larger than the F-score or the G-mean value indicates that the classifier is significantly affected by imbalanced class distribution, which is evident with the RF-based classifiers obtained from varSelRF and Boruta. However, this class imbalance problem does not apparently affect the classification performance of the RF-classifiers obtained using FPRF.10 and FPRF.20. In Lung Cancer dataset, Boruta performs better than our RF-based classifiers achieving 99% of accuracy. However, using only the first four top ranked features, the accuracy of the FPRF.4 classifier reaches 98%.

**Table 1:** Overview of the analyzed datasets.

Dataset	Sample Size	# of genes	# of classes	Class list (# of samples)
Leukemia	276	4199	6	MLL-rearrangement (21), BCR-ABL (16), T-ALL (45), TEL-AML1 (79), E2A-PBX1 (27) and Hyperdiploid>50 (88).
Lung Cancer	150	9480	4	AC1 (41), AC2 (36), SCC1(34), SCC2(39)
Psoriasis	262	9480	3	Involved-skin (91), Normal-skin (85), Uninvolved-skin (86)
PBMC	978	3700	7	Asthma quiet (521), HCV (60), Healthy (170), HIV (60), Measles (15), S. aureus (44) andSLE (108).

For each dataset, the number of samples, the number of features/genes after pre-processing the data, the number of classes and samples specified for each class are reported.

**Table 2:** Classification performance.

Method	Leukemia			Lung Cancer			Psoriasis			PBMC		
	Acc	F	G	Acc	F	G	Acc	F	G	Acc	F	G
varSelRF	0.98	0.95	0.95	0.91	0.9	0.9	0.98	0.98	0.98	0.98	0.97	0.96
Boruta	0.98	0.95	0.94	0.99	0.99	0.99	0.99	0.99	0.99	0.98	0.97	0.96
FPRF.2	0.96	0.94	0.95	0.96	0.96	0.96	0.97	0.97	0.97	0.96	0.92	0.94
FPRF.3	0.96	0.94	0.95	0.97	0.97	0.97	0.98	0.98	0.98	0.97	0.94	0.94
FPRF.4	0.97	0.96	0.96	0.98	0.98	0.98	0.98	0.98	0.98	0.98	0.95	0.96
FPRF.5	0.98	0.97	0.97	0.97	0.97	0.97	0.99	0.99	0.99	0.99	0.96	0.96
FPRF.10	0.99	0.99	0.99	0.96	0.95	0.95	0.99	0.99	0.99	0.99	0.98	0.98
FPRF.20	0.99	0.99	0.99	0.94	0.93	0.93	0.99	0.99	0.99	0.99	0.98	0.97
FPRF.30	0.98	0.98	0.98	0.92	0.92	0.92	0.99	0.99	0.99	0.99	0.98	0.97
FPRF.50	0.98	0.98	0.97	0.92	0.92	0.91	0.99	0.99	0.99	0.99	0.98	0.98
FPRF.100	0.98	0.97	0.98	0.88	0.87	0.87	0.97	0.97	0.97	0.98	0.97	0.96
FPRF.150	0.97	0.97	0.96	0.88	0.86	0.85	0.96	0.96	0.96	0.98	0.97	0.96
FPRF.200	0.97	0.96	0.96	0.87	0.85	0.85	0.96	0.95	0.95	0.98	0.97	0.96
FPRF.250	0.96	0.96	0.96	0.76	0.81	0.81	0.94	0.94	0.94	0.98	0.97	0.95

The mean accuracy values obtained over the 30 bootstrap iterations. Acc – is the overall accuracy, F – is the F-score, G – is the G-score. The highest values are highlighted in bold. NOTE: all the corresponding standard deviations are less than 0.02.

Our results confirm the recent observations of Kursa [10], where the robustness of different RF-based gene selection methods was evaluated in terms of accuracy and stability. The high accuracy of the generated gene sets is due to the nature of RF-based classifiers. They can handle a large number of noisy features without a significant increase in error. Therefore, although the classification performance is relevant for evaluating the quality of the selected feature sets, it is alone not sufficient to provide a reliable assessment of the selection quality.

### Empirical evaluation - stability

For each method, the feature stability was assessed using the sets of selected features over 30 bootstrap iterations. The stability of the different n-top ranked feature sets were compared with those obtained with varSelRF and Boruta. For each method, we identified the set of stable features by applying a recently described self-consistency-based method [10].

Table 3 reports the ratios of the self-consistent features to the total number of different features selected over the 30 bootstrap iterations for each data set and method considered. The 2, 3, 4, 5, 10 20 top-ranked features provided by our method exhibit better robustness for all data sets. This is particularly true in the case of the Lung Cancer and Psoriasis datasets, where the observed stability values are much higher than those achieved by Boruta.

**Table 3:** Selection consistency analysis.

Method	Leukemia			Lung Cancer			Psoriasis			PBMC		
	ns	tot	ns/tot	ns	tot	ns/tot	ns	tot	ns/tot	ns	tot	ns/tot
varSelRF	125	232	54%	241	1158	21%	291	734	40%	174	389	45%
Boruta	193	328	58%	20	93	22%	62	179	35%	343	667	51%
FPRF.2	4	5	80%	8	16	50%	4	2	50%	4	7	57%
FPRF.3	4	7	57%	10	22	50%	8	6	75%	5	12	42%
FPRF.4	6	12	50%	13	27	50%	9	7	78%	6	13	46%
FPRF.5	9	13	69%	13	32	41%	13	8	62%	10	14	71%
FPRF.10	17	25	68%	20	61	33%	31	20	65%	12	26	46%
FPRF.20	30	48	62%	39	126	31%	65	31	48%	29	50	58%
FPRF.30	47	78	60%	45	178	25%	44	104	42%	41	86	48%
FPRF.50	71	134	53%	84	319	26%	86	190	45%	71	136	52%
FPRF.100	130	256	51%	160	631	25%	158	554	29%	123	268	46%
FPRF.150	181	364	50%	201	914	22%	206	835	25%	185	387	48%
FPRF.200	243	541	45%	231	1166	20%	264	989	27%	232	509	46%
FPRF.250	274	619	44%	301	1328	23%	373	1082	34%	272	642	42%

The number of significantly self-consistent and all the selected genes by a given method during the 30 bootstrap iterations. *ns* – the number of significantly self-consistent genes found, *tot* – the number of different features selected over the 30 bootstrap iterations, mnsf – the mean number of selected features. The highest values are highlighted in bold.

**Figure 3:**
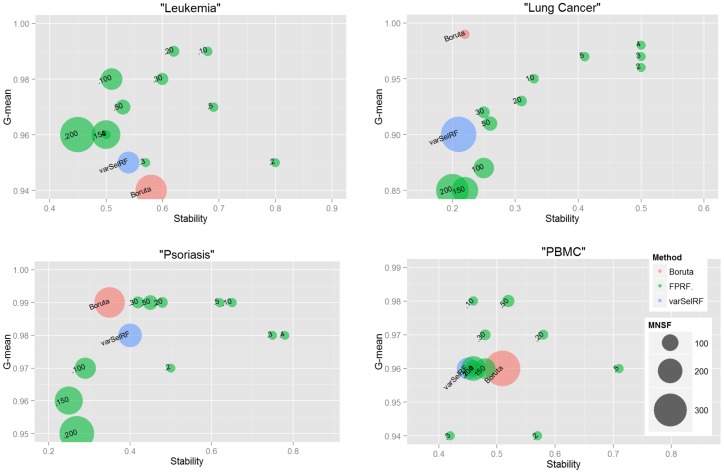
Accuracy *vs* Stability. The accuracy and the stability are evaluated together rather than individually for each data set and each analysis method. x-axis shows the percentage stability while y-axis shows the percentage accuracy (G-mean). Moreover, the size of the circles denotes the mean number of selected features over the 30 runs. Each subplot allows to analyze and identify the methods having the beset trade-off between a accuracy and stability for a specific dataset. (**.a**) trade-offs in Leukemia; (**.b**) trade-offs in Lung Cancer; (**.c**) trade-offs in Psoriasis; (**.d**) trade-offs in PBMC.

In Figure 3, we evaluated the stability and accuracy metrics together rather than individually. This figure allows to easily identify the methods providing the best trade-offs with respect to the two selected metrics. The methods providing the best trade-offs can be found in the right top corner; here there are all the methods which are strictly better on at least one metric. For instance, in the case of the Lung Cancer dataset, we can observe that Boruta provides high accuracy (99%) and very low stability (22%). While the FPRF.4 provides high accuracy (98%) and better stability (50%).

### Execution time

The average running time of the methods benchmarked in this study is reported in the Table 4. The fastest method is FPRF, when considering the sum of the execution time for both the feature selection and the prioritization steps. Instead, the slowest method is Boruta, especially for the PBMC data set, which consists of many samples. The varSelRF algorithm required less execution time than Boruta, but it is systematically slower than FPRF.

**Table 4:** Running time.

Method	Leukemia	Lung Cancer	Psoriasis	PBMC
varSelRF	5′	6′	9′	18′
Boruta	19′	20′	31′	120′
FPRF	**1**′	**2**′	**3**′	**6**′
# features	4199	9480	9480	3700
# samples	276	150	262	978

Evaluation of the running time represented as the mean over 30 bootstrap iterations. All methods investigated in this study were run single-threaded. For the proposed method the running time is compiled considering the sum of the execution times spent for the feature selection and prioritization steps.

### Biological significance of the selected feature lists

Next, we evaluated the ability of FPRF to select biologically relevant features. The lists of genes selected from each dataset are given as Data S1. Leukemia is a hematological neoplasm, in which the bone marrow generates abnormal, rapidly growing white blood cells. As expected, genes involved in acute myeloid leukemia along with other cancer-related genes, such as breast and ovarian cancers, were among the top ranked genes. Moreover, genes related to T- and B-cell activation and differentiation, as well as leukocyte activation, were also retrieved. Interestingly, FPRF selected several genes coding for proteins located in the cell membrane, suggesting that this approach might provide a valuable additional tool in leukemia differential diagnosis, which is currently dependent on flow cytometric analysis of patterns and intensity of antigen expression on the cell membrane to reach a definitive diagnosis [26]. The genes selected in the lung cancer data set are involved in epithelial differentiation. In addition, a number of known genes currently used for lung cancer subtyping were retrieved in the top positions of the ranked output list, such as KRT5 and TP63 [27].Psoriasis is a chronic inflammatory skin disease characterized by highly inflamed and sharply demarcated scaly skin lesions or plaques, which histologically show marked epidermal hyperplasia, prominent inflammatory infiltrate and increased vascularization. T-cells play a key role, and it is becoming increasingly more apparent that a pathogenic crosstalk between innate and adaptive cells underlie the dysregulated immune response that leads to abnormal epidermal proliferation [28]. As expected, the top ranked genes in the psoriasis data include members of relevant immune signaling pathways such as the IL-1 family of cytokines (IL-36G), the IFN-gamma (OASL, STAT1, CXCL1) and IL-17 (CCL20, CXCL8) signaling pathways. Furthermore, antimicrobial peptides (AMPs, S100A7A, S100A12) that bridge the immune system and the epidermal component, and kallikrein-related peptidases (KLK9) that induce AMPs were retrieved. FRPF also selected epidermal differentiation markers (members of the small proline-rich protein family (SPRR2) and the late cornified envelope family (LCE3D)), and genes associated with keratin regulation (KRT77), similar to previous studies [29], [30].Peripheral blood mononuclear cells (PBMC) represent a heterogeneous set of circulating immune cells, mainly including lymphocytes (T-, B- and NK-cells), monocytes and macrophages. The selected genes in this data set indeed highlighted relevant pathways including immune response, T-cell activation and differentiation, and host-virus interactions.

## Conclusions

We have developed a new method, FPRF, for fast feature selection and prioritization that ensures the identification of relevant and stable sets of features from high-throughput transcriptomics data. By evaluating FPRF on different multi-class microarray data sets, we show that it is able to reach a high classification power, while gaining stability over other popular algorithms.

## Supporting Information

Figure S1 **The fuzzy pattern discovery method implemented in the R package** ***DFP*** **[11]** **is described in details.**
(TIF)

Table S1 **Supplementary table reporting the values of the** ***zeta*** **and** ***piVal*** **parameters used for each analysed dataset.**
(XLSX)

Table S2 **Supplementary Table summarizing the publicly available GEO series integrated to form the PBMC dataset.**
(XLSX)

Code S1 **Supplementary methods and the implementation/evaluation in R of the FPRF method.**
(ZIP)

Data S1 **Supplementary Data containing the list of genes selected for each dataset and their ranks.**
(XLS)

## References

[1] Weinstein JN, Collisson EA, Mills GB, Shaw KRM, Ozenberger BA, et al (2013) The Cancer Genome Atlas Pan-Cancer analysis project. Nat Genet 45: 1113–1120 Available: http://www.pubmedcentral.nih.gov/articlerender.fcgi?artid=3919969&tool=pmcentrez&rendertype=abstract. 24071849 10.1038/ng.2764 PMC3919969

[2] Virtanen C, Woodgett J (2008) Clinical uses of microarrays in cancer research. Methods Mol Med 141: 87–113. 18453086 10.1007/978-1-60327-148-6_6 PMC4485473

[3] Tezak Z, Ranamukhaarachchi D, Russek-Cohen E, Gutman SI (2006) FDA perspectives on potential microarray-based clinical diagnostics. Hum Genomics 2: 236–243 10.1186/1479-7364-2-4-236 16460648 PMC3525154

[4] Saeys Y, Inza I, Larrañaga P (2007) A review of feature selection techniques in bioinformatics. Bioinformatics 23: 2507–2517. 17720704 10.1093/bioinformatics/btm344

[5] He Z, Yu W (2010) Stable feature selection for biomarker discovery. Comput Biol Chem 34: 215–225. 20702140 10.1016/j.compbiolchem.2010.07.002

[6] Abeel T, Helleputte T, Van de Peer Y, Dupont P, Saeys Y (2010) Robust biomarker identification for cancer diagnosis with ensemble feature selection methods. Bioinformatics 26: 392–398. 19942583 10.1093/bioinformatics/btp630

[7] Guyon I, Weston J, Barnhill S, Vapnik V (2002) Gene selection for cancer classification using Support Vector Machines. Mach Learn 46: 389–422 Available: http://www.pubmedcentral.nih.gov/articlerender.fcgi?artid=3740816&tool=pmcentrez&rendertype=abstract.

[8] Díaz-Uriarte R, Alvarez de Andrés S (2006) Gene selection and classification of microarray data using random forest. BMC Bioinformatics 7: 3. 16398926 10.1186/1471-2105-7-3 PMC1363357

[9] Kursa MB, Rudnicki WR (2010) Feature Selection with the Boruta Package. J Stat Softw 36: 1–13 Available: http://www.jstatsoft.org/v36/i11/paper.

[10] Kursa MB (2014) Robustness of Random Forest-based gene selection methods. BMC Bioinformatics 15: 8 Available: http://www.ncbi.nlm.nih.gov/pubmed/24410865. 24410865 10.1186/1471-2105-15-8 PMC3897925

[11] Glez-Peña D, Alvarez R, Díaz F, Fdez-Riverola F (2009) DFP: a Bioconductor package for fuzzy profile identification and gene reduction of microarray data. BMC Bioinformatics 10: 37. 19178723 10.1186/1471-2105-10-37 PMC2637236

[12] R Development Core Team (2012) R: A language and environment for statistical computing. R Foundation for Statistical Computing, Vienna, Austria. ISBN 3-900051-07-0, URL http://www.R-project.org/. R Found Stat Comput Vienna, Austria.

[13] Breiman L, Friedman JH, Olshen RA, Stone CJ (1984) Classification and Regression Trees.

[14] Hothorn T, Hornik K, Zeileis a (2006) party: A Laboratory for Recursive Part (y) itioning. R Packag version 09–0, URL http//CRAN.R-project.org.

[15] Strobl C, Zeileis A (2008) Danger: high power! - Exploring the statistical properties of a test for random forest variable importance. Univ Munich, Dep Stat Tech Rep 017: 1–8 Available: http://epub.ub.uni-muenchen.de/2111/1/techreport.pdf.

[16] Breiman L (2001) Random forests. Mach Learn 45: 5–32 10.1023/A:1010933404324

[17] Altmann A, Toloşi L, Sander O, Lengauer T (2010) Permutation importance: a corrected feature importance measure. Bioinformatics 26: 1340–1347. 20385727 10.1093/bioinformatics/btq134

[18] Strobl C, Boulesteix A-L, Zeileis A, Hothorn T (2007) Bias in random forest variable importance measures: illustrations, sources and a solution. BMC Bioinformatics 8: 25. 17254353 10.1186/1471-2105-8-25 PMC1796903

[19] Yeoh E-J, Ross ME, Shurtleff SA, Williams WK, Patel D, et al (2002) Classification, subtype discovery, and prediction of outcome in pediatric acute lymphoblastic leukemia by gene expression profiling. Cancer Cell 1: 133–143 10.1016/S1535-6108(02)00032-6 12086872

[20] Tarca AL, Lauria M, Unger M, Bilal E, Boue S, et al (2013) Strengths and limitations of microarray-based phenotype prediction: lessons learned from the IMPROVER Diagnostic Signature Challenge. Bioinformatics 29: 2892–2899 Available: http://www.ncbi.nlm.nih.gov/pubmed/23966112. 23966112 10.1093/bioinformatics/btt492 PMC3810846

[21] Irizarry RA, Hobbs B, Collin F, Beazer-Barclay YD, Antonellis KJ, et al (2003) Exploration, normalization, and summaries of high density oligonucleotide array probe level data. Biostatistics 4: 249–264. 12925520 10.1093/biostatistics/4.2.249

[22] Johnson WE, Li C, Rabinovic A (2007) Adjusting batch effects in microarray expression data using empirical Bayes methods. Biostatistics 8: 118–127. 16632515 10.1093/biostatistics/kxj037

[23] Leek JT, Johnson WE, Parker HS, Jaffe AE, Storey JD (2012) The sva package for removing batch effects and other unwanted variation in high-throughput experiments. Bioinformatics 28: 882–883 Available: http://www.pubmedcentral.nih.gov/articlerender.fcgi?artid=3307112&tool=pmcentrez&rendertype=abstract. 22257669 10.1093/bioinformatics/bts034 PMC3307112

[24] Leek JT, Storey JD (2007) Capturing heterogeneity in gene expression studies by surrogate variable analysis. PLoS Genet 3: 1724–1735. 17907809 10.1371/journal.pgen.0030161 PMC1994707

[25] Yu H, Hong S, Yang X, Ni J, Dan Y, et al (2013) Recognition of multiple imbalanced cancer types based on DNA microarray data using ensemble classifiers. Biomed Res Int 2013: 239628 Available: http://www.pubmedcentral.nih.gov/articlerender.fcgi?artid=3770038&tool=pmcentrez&rendertype=abstract. 24078908 10.1155/2013/239628 PMC3770038

[26] Jennings CD, Foon KA (1997) Recent advances in flow cytometry: application to the diagnosis of hematologic malignancy. Blood 90: 2863–2892. 9376567

[27] Mukhopadhyay S, Katzenstein A-LA (2011) Subclassification of non-small cell lung carcinomas lacking morphologic differentiation on biopsy specimens: Utility of an immunohistochemical panel containing TTF-1, napsin A, p63, and CK5/6. Am J Surg Pathol 35: 15–25 10.1097/PAS.0b013e3182036d05 21164283

[28] Di Meglio P, Perera GK, Nestle FO (2011) The Multitasking Organ: Recent Insights into Skin Immune Function. Immunity 35: 857–869. 22195743 10.1016/j.immuni.2011.12.003

[29] Suárez-Fariñas M, Li K, Fuentes-Duculan J, Hayden K, Brodmerkel C, et al (2012) Expanding the Psoriasis Disease Profile: Interrogation of the Skin and Serum of Patients with Moderate-to-Severe Psoriasis. J Invest Dermatol 132: 2552–2564 10.1038/jid.2012.184 22763790 PMC3472561

[30] Quaranta M, Knapp B, Garzorz N, Mattii M, Pullabhatla V, et al (2014) Intraindividual genome expression analysis reveals a specific molecular signature of psoriasis and eczema. Sci Transl Med 6: 244ra90–244ra90 Available: http://stm.sciencemag.org/cgi/doi/10.1126/scitranslmed.3008946. 10.1126/scitranslmed.3008946 25009230

