# Supplementary figures and images for: A Robust and Accurate Method for Feature Selection and Prioritization from Multi-Class OMICs Data
Source: PLoS One. 2014 Sep 23;9(9):e107801. doi: 10.1371/journal.pone.0107801 (PMC4172658; doi:10.1371/journal.pone.0107801)

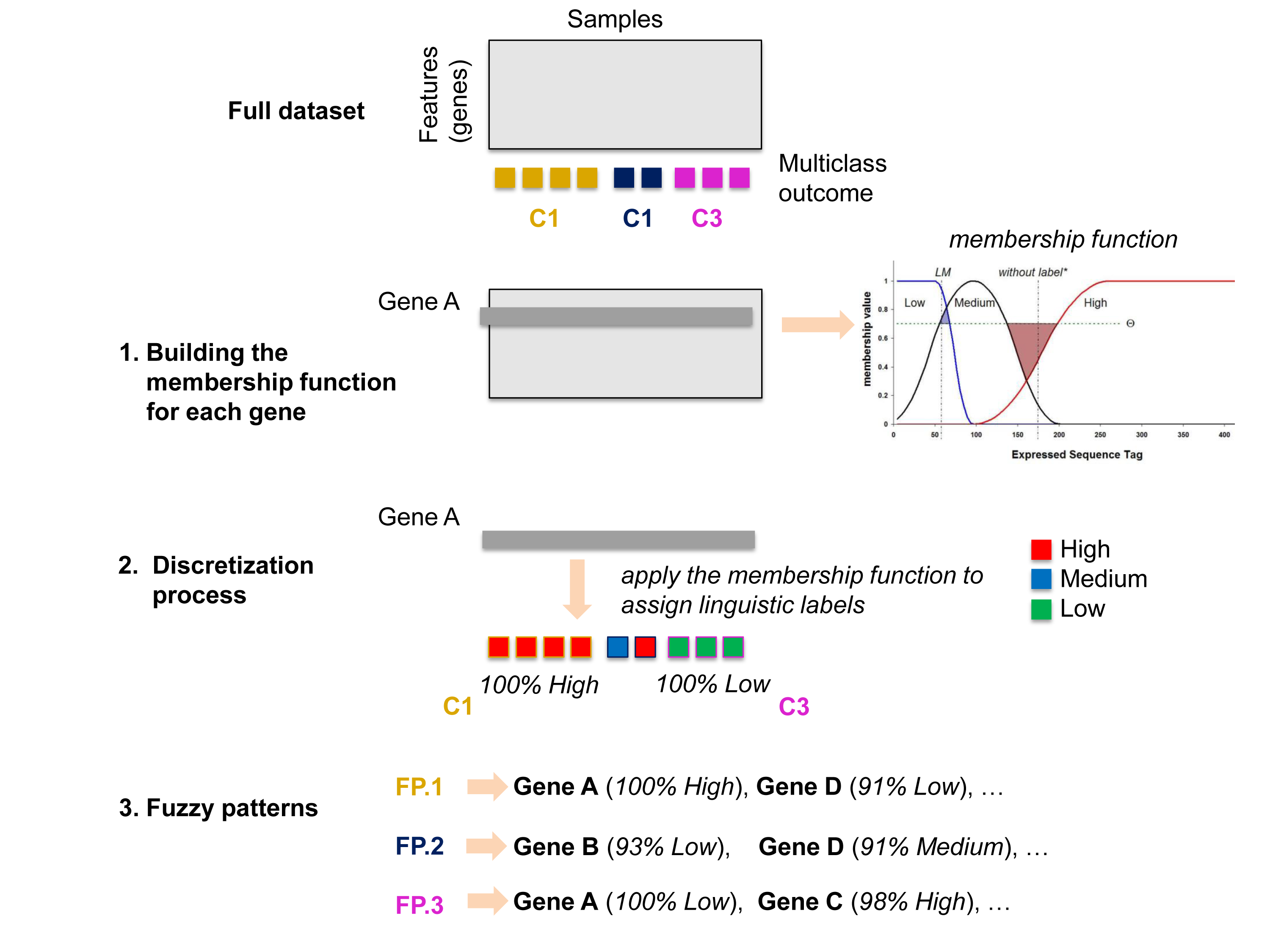

Supplement: Figure S1 — The fuzzy pattern discovery method implemented in the R package DFP [11] is described in details. (TIF) [file pone.0107801.s001.tif]
