# Supplementary material for: A Robust and Accurate Method for Feature Selection and Prioritization from Multi-Class OMICs Data
Source: PLoS One. 2014 Sep 23;9(9):e107801. doi: 10.1371/journal.pone.0107801 (PMC4172658; doi:10.1371/journal.pone.0107801)
Supplement: Code S1 — Supplementary methods and the implementation/evaluation in R of the FPRF method. (ZIP) [file pone.0107801.s004.zip › FPRF_scripts/source_mod_party/party/inst/doc/MOB.pdf]

party with the `mob`: Model-Based Recursive Partitioning in R

Achim Zeileis
Universität Innsbruck

Torsten Hothorn
Ludwig-Maximilians-Universität München

Kurt Hornik
WU Wirtschafts-universität Wien

Abstract

The **party** package (Hothorn, Hornik, and Zeileis 2006) provides the function `mob()` implementing a recently suggested algorithm for model-based recursive partitioning (Zeileis, Hothorn, and Hornik 2008). The basic steps are: (1)~fit a parametric model to a data set, (2)~test for parameter instability over a set of partitioning variables, (3)~if there is some overall parameter instability, split the model with respect to the variable associated with the highest instability, (4)~repeat the procedure in each of the child nodes. It is discussed how these steps of the conceptual algorithm are translated into computational tools in an object-oriented manner, allowing the user to plug in various types of parametric models. The outcome is a tree where each node is associated with a fitted parametric model that can be effectively visualized and summarized.

Keywords:~parametric models, object-orientation, recursive partitioning.

1. Motivation

Consider a parametric model $\mathcal{M}(Y, \theta)$ with (possibly vector-valued) observations Y and a k-dimensional vector of parameters θ. This model could be a (possibly multivariate) normal distribution for Y, or some kind of regression model when $Y = (y, x)$ can be split up into a dependent variable y and regressors x. An example for the latter could be a linear regression model $y = x^\top \theta$ or a generalized linear model (GLM) or a survival regression.

Given n observations Y_i ($i = 1, \dots, n$) the model can be fitted by minimizing some objective function $\Psi(Y, \theta)$, e.g., a residual sum of squares or a negative log-likelihood leading to ordinary least squares (OLS) or maximum likelihood (ML) estimation.

If a global model for all n observations does not fit well and further covariates $Z_1, \dots, Z_\ell$ are available, it might be possible to partition the n observations with respect to these variables and find a fitting model in each cell of the partition. The algorithm described here tries to find such a partition adaptively using a greedy forward search. This procedure is implemented in the function `mob()` and described in more detail in the following section. However, we we will state some goals and design principles in advance.

To translate the model-based partitioning problem into R, we start with a formula description of the variables involved. This formula should be of type `y ~ x1 + ... + xk | z1 + ... + zl` where the variables on the left of the `|` specify the data Y and the variables on the right specify the partitioning variables Z_j. Classical regression trees usually have a univariate response Y

and various partitioning variables, i.e., could be specified as `y ~ 1 | z1 + ... + zl`. Structural change models, on the other hand, are usually regression models that are segmented with respect to a single partitioning variable, typically time: `y ~ x1 + ... + xk | z`.

The type of models $\mathcal{M}$ to be used with `mob()` should not be confined (by the implementation), hence we have written an object-oriented implementation. The idea is that $\mathcal{M}$ is translated into software by a model of class "`StatModel`" as provided by the **modeltools** package. The algorithm the relies on various methods being available for these models. The "`StatModel`" objects `linearModel` and `glinearModel`, implementing (generalized) linear regression models, are readily available in **modeltools**, others can easily be user-defined.

2. The model-based recursive partitioning algorithm

The basic idea is to grow a tee in which every node is associated with a model of type $\mathcal{M}$. To assess whether splitting of the node is necessary a fluctuation test for parameter instability is performed. If there is significant instability with respect to any of the partitioning variables Z_j, the node is splitted into B locally optimal segments (currently only $B = 2$ is implemented) and then the procedure is repeated in each of the B children. If no more significant instabilities can be found, the recursion stops. More precisely, the steps of the algorithm are

1. Fit the model once to all observations in the current node.
2. Assess whether the parameter estimates are stable with respect to every partitioning variable $Z_1, \dots, Z_\ell$. If there is some overall instability, select the variable Z_j associated with the highest parameter instability, otherwise stop.
3. Compute the split point(s) that locally optimize the objective function Ψ.
4. Split the node into child nodes and repeat the procedure.

The details for steps 1–3 are specified in the following.

2.1. Parameter estimation

This step of the algorithm is common practice, the only additional requirement is (as previously noted) that model has to be of the class "`StatModel`" as provided by **modeltools**. Looking at the source code for the `linearModel` provided by this package illustrates how a simple wrapper to existing R functionality can be written. In particular, a method to the generic function `reweight()` has to be available. The reason is that it is inefficient to fit a brand-new model `modelobj` (including formula-parsing) in every node—much computation time is saved if simply `reweight(modelobj, weights)` is called in each of the child nodes. The `weights` argument controls which observations go into which of the child nodes.

2.2. Testing for parameter instability

The task in this step of the algorithm is to find out whether the parameters of the fitted model are stable over each particular ordering implied by the partitioning variables Z_j or whether splitting the sample with respect to one of the Z_j might capture instabilities in the parameters

and thus improve the fit. The tests used in this step belong to the class of generalized M-fluctuation tests (Zeileis and Hornik 2007; Zeileis 2005). For numerical partitioning variables Z_j the sup LM~statistic is used which is the maximum over all single split LM statistics. For categorical partitioning variables, a χ^2~statistic is employed which captures the fluctuation within each of the categories of Z_j.

For computing the test statistics and corresponding p~values p_j for each of the partitioning variables Z_j in R, the only requirement is that there are methods for the extractor functions `estfun()` and `weights()`. The `estfun()` method extracts the empirical estimating functions (model scores) from the fitted `modelobj`, these are the main ingredient for M-fluctuation tests. The `weights()` method is used to determine which observations are in the current node (i.e., have a weight greater than zero) and which are not (i.e., have zero weight).

To determine whether there is some overall instability, it is checked whether the minial p~value $p_{j^*} = \min_{j=1,\dots,\ell} p_j$ falls below a pre-specified significance level α (by default $\alpha = 0.05$) or not. To adjust for multiple testing, the p values can be Bonferroni adjusted (which is the default). If there is significant instability, the variable Z_{j^*} associated with the minimal p~value is used for splitting the node.

2.3. Splitting

In this step, the observation in the current node are split with respect to the chosen partitioning variable Z_{j^*} into B child nodes. Currently, the infrastructure in **party** only supports binary splits, i.e., $B = 2$. For deterimining the split point, an exhaustive search procedure is adopted: For each conceivable split point, the B child node models are fit and the split associated with the minimal value of the objective function Ψ is chosen.

Computationally, this means that the fitted model `modelobj` is `reweight()`ed for each of the child nodes. The observations entering a child node keep their current weight while those observations that go into different child nodes receive zero weight. To compare the objective function Ψ, an extractor function is required to compute it from the fitted `modelobj`. This extractor function can be user-specified and set in `mob_control()`, it defaults to `deviance()`.

This concludes one iteration of the recursive partitioning algorithm and steps~1–3 are carried out again in each of the B daughter nodes until no significant instability is detected in step~2.

3. Illustrations

3.1. Boston housing data

Since the analysis by Breiman and Friedman (1985), the Boston housing data are a popular and well-investigated empirical basis for illustrating non-linear regression methods both in machine learning and statistics (see Gama 2004; Samarov, Spokoiny, and Vial 2005, for two recent examples) and we follow these examples by segmenting a bivariate linear regression model for the house values.

Thus, the model $\mathcal{M}$ used is `linearModel` from the **modeltools** package which is automatically loaded together with **party**.

```
> library("party")
```

The data set is available in package **mlbench** via

```
> data("BostonHousing", package = "mlbench")
```

and provides $n = 506$ observations of the median value of owner-occupied homes in Boston (in USD˜1000) along with 14 covariates including in particular the number of rooms per dwelling (`rm`) and the percentage of lower status of the population (`lstat`). A segment-wise linear relationship between the value and these two variables is very intuitive, whereas the shape of the influence of the remaining covariates is rather unclear and hence should be learned from the data. Therefore, a linear regression model for median value explained by `rm^2` and `log(lstat)` with $k = 3$ regression coefficients is employed and partitioned with respect to all $\ell = 11$ remaining variables. To facilitate subsequent commands, the transformations are explicitely stored in `BostonHousing`:

```
> BostonHousing$lstat <- log(BostonHousing$lstat)
> BostonHousing$rm <- BostonHousing$rm^2
```

Choosing appropriate transformations of the dependent variable and the regressors that enter the linear regression model is important to obtain a well-fitting model in each segment and we follow in our choice the recommendations of Breiman and Friedman (1985). Monotonous transformations of the partitioning variables do not affect the recursive partitioning algorithm and hence do not have to be performed. However, it is important to distinguish between numerical and categorical variables for choosing an appropriate parameter stability test. The variable `chas` is a dummy indicator variable (for tract bounds with Charles river) and should thus be turned into a factor. Furthermore, the variable `rad` is an index of accessibility to radial highways and takes only 9 distinct values. Thus it is most appropriately treated as an ordered factor.

```
> BostonHousing$chas <- factor(BostonHousing$chas, levels = 0:1, labels = c("no", "yes"))
> BostonHousing$rad <- factor(BostonHousing$rad, ordered = TRUE)
```

Both transformations only affect the parameter stability test chosen (step˜2), not the splitting procedure (step˜3).

The model is estimated by OLS, the instability is assessed using a Bonferroni-corrected significance level of $\alpha = 0.05$ and the nodes are split with a required minimal segment size of 40 observations. The control parameters are thus set to

```
> ctrl <- mob_control(alpha = 0.05, bonferroni = TRUE, minsplit = 40,
+   objfun = deviance, verbose = TRUE)
```

Actually, all of these settings are the defaults except `minsplit = 40` and `verbose = TRUE` which causes some information about the fitting process being written to the screen. The objective function `deviance()` extracts in this case the residual sum of squares from a fitted `linearModel` object.

Having collected all building blocks, we can now call the function `mob()` that takes the model specification of the linear regression model `medv ~ lstat + rm` plus all partitioning variables, along with the `data` set, the `control` settings and the `model` to be used.

```
> fmBH <- mob(medv ~ lstat + rm | zn + indus + chas + nox + age + dis + rad + tax + crim +
+   data = BostonHousing, control = ctrl, model = linearModel)

-------------------------------------------
Fluctuation tests of splitting variables:
                    zn        indus         chas          nox          age
statistic 3.363356e+01 6.532322e+01 2.275635e+01 8.136281e+01 3.675850e+01
p.value   1.023987e-04 1.363602e-11 4.993053e-04 3.489797e-15 2.263798e-05
                   dis          rad          tax         crim            b
statistic 6.848533e+01 1.153641e+02 9.068440e+01 8.655065e+01 3.627629e+01
p.value   2.693904e-12 7.087680e-13 2.735524e-17 2.356348e-16 2.860686e-05
               ptratio
statistic 7.221524e+01
p.value   3.953623e-13

Best splitting variable: tax
Perform split? yes
-------------------------------------------

Node properties:
tax <= 432; criterion = 1, statistic = 115.364

-------------------------------------------
Fluctuation tests of splitting variables:
                    zn      indus      chas          nox        age
statistic 27.785009791 21.3329346 8.0272421 23.774323202 11.9204284
p.value    0.001494064  0.0285193 0.4005192  0.009518732  0.7666366
                   dis          rad          tax         crim         b
statistic 24.268011081 50.481593270 3.523250e+01 3.276813e+01 9.0363245
p.value    0.007601532  0.003437763 4.275527e-05 1.404487e-04 0.9871502
               ptratio
statistic 4.510680e+01
p.value   3.309747e-07

Best splitting variable: ptratio
Perform split? yes
-------------------------------------------

Node properties:
ptratio <= 15.2; criterion = 1, statistic = 50.482

-------------------------------------------
Fluctuation tests of splitting variables:
                    zn       indus        chas         nox         age
statistic 3.233350e+01 22.26864036 12.93407112 22.10510234 20.41295354
p.value   1.229678e-04  0.01504788  0.05259509  0.01622098  0.03499731
                 dis          rad          tax        crim           b
```

```
statistic 17.7204735 5.526565e+01 2.879128e+01 20.28503194 6.5549665
p.value    0.1091769 7.112214e-04 6.916307e-04  0.03706934 0.9999522
               ptratio
statistic 4.789850e+01
p.value   4.738855e-08

Best splitting variable: ptratio
Perform split? yes
-------------------------------------------

Node properties:
ptratio <= 19.6; criterion = 1, statistic = 55.266

-------------------------------------------
Fluctuation tests of splitting variables:
                 zn      indus       chas        nox       age        dis
statistic 14.971474 14.6477733 7.1172962 14.3455158 8.2176363 16.1112185
p.value    0.280361  0.3134649 0.5405005  0.3467974 0.9906672  0.1847818
                  rad          tax     crim         b    ptratio
statistic 43.17824350 3.447271e+01 9.340075 8.7773142 10.8469969
p.value    0.03281124 4.281939e-05 0.952996 0.9772696  0.8202694

Best splitting variable: tax
Perform split? yes
-------------------------------------------

Node properties:
tax <= 265; criterion = 1, statistic = 43.178

-------------------------------------------
Fluctuation tests of splitting variables:
                 zn     indus     chas       nox        age       dis
statistic 11.998039 7.3971233 7.227770 9.2936189 14.3023962 8.9239826
p.value    0.574642 0.9931875 0.522447 0.9119621  0.2886603 0.9389895
                 rad        tax       crim         b    ptratio
statistic 33.1746444 16.6666129 11.7143758 9.9050903 11.5927528
p.value    0.3926249  0.1206412  0.6153455 0.8539893  0.6328381

Best splitting variable: tax
Perform split? no
-------------------------------------------

-------------------------------------------
Fluctuation tests of splitting variables:
                  zn     indus         chas         nox       age        dis
statistic 10.9187926 9.0917078 2.754081e+01 17.39203006 4.6282349 11.9581600
p.value    0.7091039 0.9172303 4.987667e-05  0.08922543 0.9999992  0.5607267
```

```
                rad        tax     crim        b   ptratio
statistic 0.2557803 10.9076165 3.711175 3.158329 9.8865054
p.value   1.0000000  0.7106612 1.000000 1.000000 0.8410064

Best splitting variable: chas
Perform split? yes
-------------------------------------------

Splitting factor variable, objective function:
 no
Inf

No admissable split found in 'chas'
```

The result is the fitted model `fmBH` of class "`mob`" that contains the tree with a fitted linear regression associated with every node. Printing this object will show the splits, their p values and call the `print()` method for the model in each terminal node (i.e., this simply relies on a `print()` method being available for the fitted model and re-uses it).

```
> fmBH

1) tax <= 432; criterion = 1, statistic = 115.364
  2) ptratio <= 15.2; criterion = 1, statistic = 50.482
    3)*  weights = 72
Terminal node model
Linear model with coefficients:
(Intercept)       lstat          rm
     9.2349     -4.9391      0.6859

  2) ptratio > 15.2
    4) ptratio <= 19.6; criterion = 1, statistic = 55.266
      5) tax <= 265; criterion = 1, statistic = 43.178
        6)*  weights = 63
Terminal node model
Linear model with coefficients:
(Intercept)       lstat          rm
     3.9637     -2.7663      0.6881

      5) tax > 265
        7)*  weights = 162
Terminal node model
Linear model with coefficients:
(Intercept)       lstat          rm
    -1.7984     -0.2677      0.6539

    4) ptratio > 19.6
      8)*  weights = 56
```

```
Terminal node model
Linear model with coefficients:
(Intercept)       lstat          rm
    17.5865     -4.6190      0.3387

1) tax > 432
  9)*  weights = 153
Terminal node model
Linear model with coefficients:
(Intercept)       lstat          rm
    68.2971    -16.3540     -0.1478
```

Looking at the printed output is typically rather tedious, a visualization via the `plot()` method

```
> plot(fmBH)
```

is much easier to interpret. By default, this produces partial scatter plots of the variable y against each of the regressors x_i in the terminal nodes. Each scatter plot also shows the fitted values, i.e., a project of the fitted hyperplane.

From this visualization, it can be seen that in the nodes˜4, 6, 7 and 8 the increase of value with the number of rooms dominates the picture (upper panel) whereas in node˜9 the decrease with the lower status population percentage (lower panel) is more pronounced. Splits are performed in the variables `tax` (poperty-tax rate) and `ptratio` (pupil-teacher ratio).

Various quantities of interest can be computed, provided that the `model` used provides the corresponding methods, e.g., `predict()`, `residuals()`, `logLik()`, `coef()` and `summary()`. The latter two by default try to extract information for the terminal nodes, but a `node` argument can be set to the node IDs of interest. As an example, the regression coefficients for the terminal node models can be easily extracted by

```
> coef(fmBH)

  (Intercept)      lstat         rm
3    9.234880  -4.939096  0.6859136
6    3.963720  -2.766287  0.6881287
7   -1.798387  -0.267707  0.6538864
8   17.586490  -4.618975  0.3386744
9   68.297087 -16.354006 -0.1477939
```

reflecting the differences of the models that can also be seen in the the associated `plot()`. Even more information is available in a `summary()`, e.g., for node 7:

```
> summary(fmBH, node = 7)

Call:
NULL
```

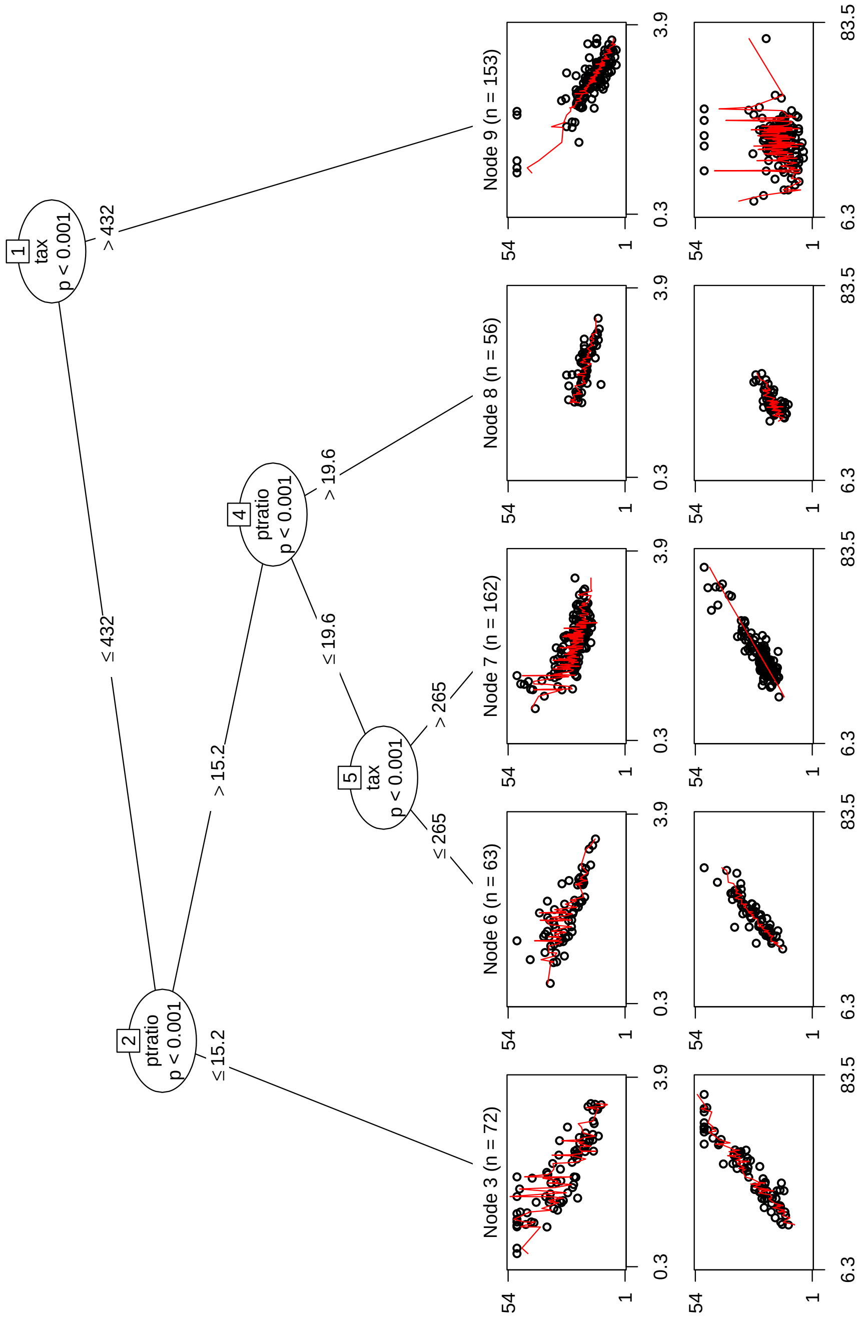

Figure 1: Linear-regression-based tree for the Boston housing data. The plots in the leaves give partial scatter plots for `rm` (upper panel) and `lstat` (lower panel).

```
Weighted Residuals:
   Min     1Q Median     3Q    Max 
-9.092  0.000  0.000  0.000 10.236 

Coefficients:
            Estimate Std. Error t value Pr(>|t|)    
(Intercept) -1.79839    2.84702  -0.632    0.529    
lstat       -0.26771    0.69581  -0.385    0.701    
rm           0.65389    0.03757  17.404   <2e-16 ***
---
Signif. codes:  0 '***' 0.001 '**' 0.01 '*' 0.05 '.' 0.1 ' ' 1 

Residual standard error: 2.652 on 159 degrees of freedom
Multiple R-squared:  0.8173,	Adjusted R-squared:  0.815 
F-statistic: 355.6 on 2 and 159 DF,  p-value: < 2.2e-16
```

The test statistics and p~values computed in each node, can be extracted analogously by using the method for the function `sctest()` (for performing structural change tests).

```
> sctest(fmBH, node = 7)

                 zn     indus     chas       nox       age       dis
statistic 11.998039 7.3971233 7.227770 9.2936189 14.3023962 8.9239826
p.value    0.574642 0.9931875 0.522447 0.9119621  0.2886603 0.9389895
                 rad        tax       crim         b    ptratio
statistic 33.1746444 16.6666129 11.7143758 9.9050903 11.5927528
p.value    0.3926249  0.1206412  0.6153455 0.8539893  0.6328381
```

For summarizing the quality of the fit, we could compute the mean squared error, log-likelihood or AIC:

```
> mean(residuals(fmBH)^2)

[1] 12.03518

> logLik(fmBH)

'log Lik.' -1310.506 (df=24)

> AIC(fmBH)

[1] 2669.013
```

As the `logLik()` method simply re-uses the method for `linearModel` objects, this does not only report 19 estimated parameters (3 parameters in each of the 5 terminal nodes plus $5 - 1$

split points) but 24 parameters because each terminal node is additionally associated with a variance estimate. However, for the fitting process, the variance was treated as a nuisance parameter as we employed OLS estimation (rather than fully-specified ML estimation).

3.2. Pima Indians diabetes data

Another popular benchmark data set for binary classifications is the Pima Indians diabetes database which is also available from **mlbench**:

```
> data("PimaIndiansDiabetes2", package = "mlbench")
> PimaIndiansDiabetes <- na.omit(PimaIndiansDiabetes2[,-c(4, 5)])
```

After omitting missing values (and the variables `triceps` and `insulin` which are missing for most women), the data set provides diabetes test results for $n = 724$ women along with 7 covariates including in particular the plasma glucose concentration `glucose` as an important predictor for diabetes. Fitting a logistic regression model `diabetes ~ glucose` seems to be straightforward, whereas the influence of the remaining variables should again be learned by recursive partitioning. This will yield a model tree with $k = 2$ regression coefficients in each terminal node, partitioned with respect to the remaining $\ell = 5$ remaining variables.

The model is estimated by ML employing the `glinearModel`, the instability is assessed using a Bonferroni-corrected significance level of $\alpha = 0.05$ and the nodes are split with a required minimal segment size of 20 observations. Hence, all control parameters correspond to the default values in `mob_control()` and do not have to be set explicitely in the `mob()` call:

```
> fmPID <- mob(diabetes ~ glucose | pregnant + pressure + mass + pedigree + age,
+   data = PimaIndiansDiabetes, model = glinearModel, family = binomial())
```

To visualize this, we simply call again:

```
> plot(fmPID)
```

which produces again a plot of the dependent variable y against the only regressors x in the terminal nodes. As y is `diabetes`, a binary variable, and x is `glucose`, a numeric variable, a spinogram is chosen for visualization. The breaks in the spinogram are the five-point summary of `glucose` on the full data set. The fitted lines are the mean predicted probabilities in each group.

The model tree distinguishes three different groups:

#2 Women with low body mass index that have on average a low risk of diabetes, however this increases clearly with glucose level.

#4 Women with average and high body mass index, younger than 30 years, that have a higher avarage risk that also increases with glucose level.

#5 Women with average and high body mass index, older than 30 years, that have a high avarage risk that increases only slowly with glucose level.

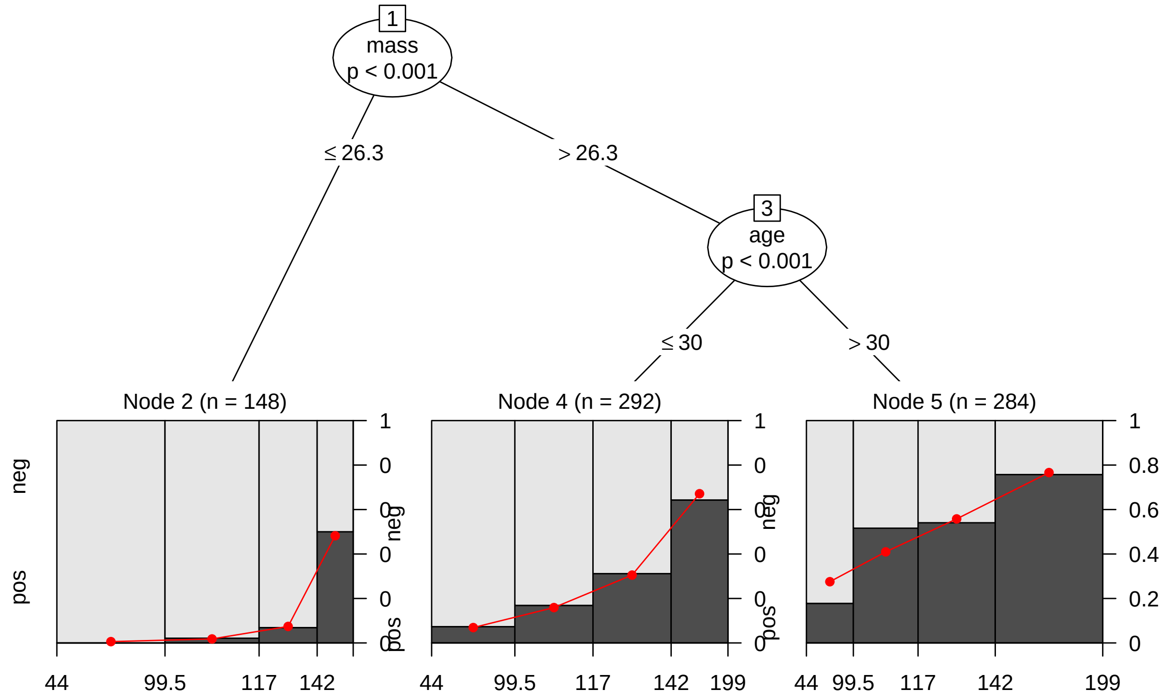

Figure 2: Logistic-regression-based tree for the Pima Indians diabetes data. The plots in the leaves give spinograms for `diabetes` versus `glucose`.

The same interpretation can also be drawn from the coefficient estimates and the corresponding odds ratios (with respect to glucose):

```
> coef(fmPID)

  (Intercept)    glucose
2  -10.999447 0.06456780
4   -6.573067 0.04504490
5   -3.318569 0.02748038

> exp(coef(fmPID)[,2])

       2        4        5
1.066698 1.046075 1.027861
```

i.e., the odds increase by 6.7%, 4.6% and 2.8% with respect to glucose in the three groups.

4. Conclusion

The function `mob()` in the **party** package provides a flexible and object-oriented implementation of the general algorithm for model-based recursive partitioning. Models of class "`StatModel`"—that employ a formula interface and are equipped with methods for the generic functions `reweight()`, `weights()`, `estfun()` plus some function for extracting the value of the objective function—can be easily partitioned. The resulting "`mob`" tree can be flexibly summarized, both numerically and graphically, and used for predictions on new data.

References

Breiman L, Friedman JH (1985). "Estimating Optimal Transformations for Multiple Regression and Correlation." *Journal of the American Statistical Association*, **80**(391), 580–598.

Gama J (2004). "Functional Trees." *Machine Learning*, **55**, 219–250.

Hothorn T, Hornik K, Zeileis A (2006). "Unbiased Recursive Partitioning: A Conditional Inference Framework." *Journal of Computational and Graphical Statistics*, **15**(3), 651–674. `doi:10.1198/106186006X133933`.

Samarov A, Spokoiny V, Vial C (2005). "Component Indentification and Estimation in Nonlinear High-Dimension Regression Models by Structural Adaptation." *Journal of the American Statistical Association*, **100**(470), 429–445.

Zeileis A (2005). "A Unified Approach to Structural Change Tests Based on ML Scores, F Statistics, and OLS Residuals." *Econometric Reviews*, **24**, 445–466.

Zeileis A, Hornik K (2007). "Generalized M-Fluctuation Tests for Parameter Instability." *Statistica Neerlandica*, **61**(4), 488–508. `doi:10.1111/j.1467-9574.2007.00371.x`.

Zeileis A, Hothorn T, Hornik K (2008). "Model-Based Recursive Partitioning." *Journal of Computational and Graphical Statistics*, **17**(2), 492–514. `doi:10.1198/106186008X319331`.

Affiliation:

Achim Zeileis
Department of Statistics
Faculty of Economics and Statistics
Universität Innsbruck
Universitätsstr.~15
6020 Innsbruck, Austria
E-mail: `Achim.Zeileis@R-project.org`
URL: `http://eeecon.uibk.ac.at/~zeileis/`

Torsten Hothorn
Institut für Statistik
Ludwig-Maximilians-Universität München
Ludwigstraße 33
80539 München, Germany
E-mail: `Torsten.Hothorn@R-project.org`
URL: `http://www.stat.uni-muenchen.de/~hothorn/`

Kurt Hornik
Institute for Statistics and Mathematics
WU Wirtschaftsuniversität Wien
Augasse 2–6
1090 Wien, Austria
E-mail: `Kurt.Hornik@R-project.org`
URL: `http://statmath.wu.ac.at/~hornik/`
