# Supplementary material for: A Robust and Accurate Method for Feature Selection and Prioritization from Multi-Class OMICs Data
Source: PLoS One. 2014 Sep 23;9(9):e107801. doi: 10.1371/journal.pone.0107801 (PMC4172658; doi:10.1371/journal.pone.0107801)
Supplement: Code S1 — Supplementary methods and the implementation/evaluation in R of the FPRF method. (ZIP) [file pone.0107801.s004.zip › FPRF_scripts/source_mod_party/party/inst/doc/party.pdf]

party: A Laboratory for Recursive Partytioning

Torsten Hothorn
Ludwig-Maximilians-Universität München

Kurt Hornik
Wirtschaftsuniversität Wien

Achim Zeileis
Wirtschaftsuniversität Wien

Abstract

The **party** package (Hothorn, Hornik, and Zeileis 2006) aims at providing a recursive part(y)itioning laboratory assembling various high- and low-level tools for building tree-based regression and classification models. This includes conditional inference trees (`ctree`), conditional inference forests (`cforest`) and parametric model trees (`mob`). At the core of the package is `ctree`, an implementation of conditional inference trees which embed tree-structured regression models into a well defined theory of conditional inference procedures. This non-parametric class of regression trees is applicable to all kinds of regression problems, including nominal, ordinal, numeric, censored as well as multivariate response variables and arbitrary measurement scales of the covariates. This vignette comprises a practical guide to exploiting the flexible and extensible computational tools in **party** for fitting and visualizing conditional inference trees.

Keywords:~conditional inference, non-parametric models, recursive partitioning.

1. Introduction

The majority of recursive partitioning algorithms are special cases of a simple two-stage algorithm: First partition the observations by univariate splits in a recursive way and second fit a constant model in each cell of the resulting partition. The most popular implementations of such algorithms are 'CART' (Breiman, Friedman, Olshen, and Stone 1984) and 'C4.5' (Quinlan 1993). Not unlike AID, both perform an exhaustive search over all possible splits maximizing an information measure of node impurity selecting the covariate showing the best split. This approach has two fundamental problems: overfitting and a selection bias towards covariates with many possible splits. With respect to the overfitting problem Mingers (1987) notes that the algorithm

> [...] has no concept of statistical significance, and so cannot distinguish between a significant and an insignificant improvement in the information measure.

With the **party** package (see Hothorn *et*~*al.* 2006, for a full description of its methodological foundations) we enter at the point where White and Liu (1994) demand for

> [...] a *statistical* approach [to recursive partitioning] which takes into account the *distributional* properties of the measures.

We present a unified framework embedding recursive binary partitioning into the well defined theory of permutation tests developed by Strasser and Weber (1999). The conditional distri-

bution of statistics measuring the association between responses and covariates is the basis for an unbiased selection among covariates measured at different scales. Moreover, multiple test procedures are applied to determine whether no significant association between any of the covariates and the response can be stated and the recursion needs to stop.

2. Recursive binary partitioning

We focus on regression models describing the conditional distribution of a response variable $\mathbf{Y}$ given the status of m covariates by means of tree-structured recursive partitioning. The response $\mathbf{Y}$ from some sample space $\mathcal{Y}$ may be multivariate as well. The m-dimensional covariate vector $\mathbf{X} = (X_1, \dots, X_m)$ is taken from a sample space $\mathcal{X} = \mathcal{X}_1 \times \cdots \times \mathcal{X}_m$. Both response variable and covariates may be measured at arbitrary scales. We assume that the conditional distribution $D(\mathbf{Y}|\mathbf{X})$ of the response $\mathbf{Y}$ given the covariates $\mathbf{X}$ depends on a function f of the covariates

$$D(\mathbf{Y}|\mathbf{X}) = D(\mathbf{Y}|X_1, \dots, X_m) = D(\mathbf{Y}|f(X_1, \dots, X_m)),$$

where we restrict ourselves to partition based regression relationships, i.e., r disjoint cells $B_1, \dots, B_r$ partitioning the covariate space $\mathcal{X} = \bigcup_{k=1}^{r} B_k$. A model of the regression relationship is to be fitted based on a learning sample $\mathcal{L}_n$, i.e., a random sample of n independent and identically distributed observations, possibly with some covariates X_{ji} missing,

$$\mathcal{L}_n \quad = \quad \{(\mathbf{Y}_i, X_{1i}, \dots, X_{mi}); i = 1, \dots, n\}.$$

For the sake of simplicity, we use a learning sample

```
> ls <- data.frame(y = gl(3, 50, labels = c("A", "B", "C")), x1 = rnorm(150) + rep(c(1, 0,
+ 50)), x2 = runif(150))
```

in the following illustrations. A generic algorithm for recursive binary partitioning for a given learning sample $\mathcal{L}_n$ can be formulated using non-negative integer valued case weights $\mathbf{w} = (w_1, \dots, w_n)$. Each node of a tree is represented by a vector of case weights having non-zero elements when the corresponding observations are elements of the node and are zero otherwise. The following algorithm implements recursive binary partitioning:

1. For case weights $\mathbf{w}$ test the global null hypothesis of independence between any of the m covariates and the response. Stop if this hypothesis cannot be rejected. Otherwise select the covariate X_{j^*} with strongest association to $\mathbf{Y}$.

2. Choose a set $A^* \subset \mathcal{X}_{j^*}$ in order to split $\mathcal{X}_{j^*}$ into two disjoint sets A^* and $\mathcal{X}_{j^*} \setminus A^*$. The case weights $\mathbf{w}_{\text{left}}$ and $\mathbf{w}_{\text{right}}$ determine the two subgroups with $w_{\text{left},i} = w_i I(X_{j^*i} \in A^*)$ and $w_{\text{right},i} = w_i I(X_{j^*i} \notin A^*)$ for all $i = 1, \dots, n$ ($I(\cdot)$ denotes the indicator function).

3. Recursively repeat steps 1 and 2 with modified case weights $\mathbf{w}_{\text{left}}$ and $\mathbf{w}_{\text{right}}$, respectively.

The separation of variable selection and splitting procedure into steps 1 and 2 of the algorithm is the key for the construction of interpretable tree structures not suffering a systematic tendency towards covariates with many possible splits or many missing values. In addition,

a statistically motivated and intuitive stopping criterion can be implemented: We stop when the global null hypothesis of independence between the response and any of the m covariates cannot be rejected at a pre-specified nominal level~α. The algorithm induces a partition $\{B_1, \dots, B_r\}$ of the covariate space $\mathcal{X}$, where each cell $B \in \{B_1, \dots, B_r\}$ is associated with a vector of case weights.

In package **party**, the dependency structure and the variables may be specified in a traditional formula based way

```
> library("party")
> ctree(y ~ x1 + x2, data = ls)
```

Case counts $\mathbf{w}$ may be specified using the `weights` argument.

3. Recursive partitioning by conditional inference

In the main part of this section we focus on step 1 of the generic algorithm. Unified tests for independence are constructed by means of the conditional distribution of linear statistics in the permutation test framework developed by Strasser and Weber (1999). The determination of the best binary split in one selected covariate and the handling of missing values is performed based on standardized linear statistics within the same framework as well.

3.1. Variable selection and stopping criteria

At step 1 of the generic algorithm given in Section~2 we face an independence problem. We need to decide whether there is any information about the response variable covered by any of the m covariates. In each node identified by case weights $\mathbf{w}$, the global hypothesis of independence is formulated in terms of the m partial hypotheses $H_0^j : D(\mathbf{Y}|X_j) = D(\mathbf{Y})$ with global null hypothesis $H_0 = \bigcap_{j=1}^m H_0^j$. When we are not able to reject H_0 at a pre-specified level α, we stop the recursion. If the global hypothesis can be rejected, we measure the association between $\mathbf{Y}$ and each of the covariates $X_j, j = 1, \dots, m$, by test statistics or P-values indicating the deviation from the partial hypotheses H_0^j.

For notational convenience and without loss of generality we assume that the case weights w_i are either zero or one. The symmetric group of all permutations of the elements of $(1, \dots, n)$ with corresponding case weights $w_i = 1$ is denoted by $S(\mathcal{L}_n, \mathbf{w})$. A more general notation is given in the Appendix. We measure the association between $\mathbf{Y}$ and $X_j, j = 1, \dots, m$, by linear statistics of the form

$$\mathbf{T}_j(\mathcal{L}_n, \mathbf{w}) = \mathrm{vec}\left(\sum_{i=1}^n w_i g_j(X_{ji}) h(\mathbf{Y}_i, (\mathbf{Y}_1, \dots, \mathbf{Y}_n))^\top\right) \in \mathbb{R}^{p_j q} \quad (1)$$

where $g_j : \mathcal{X}_j \to \mathbb{R}^{p_j}$ is a non-random transformation of the covariate X_j. The transformation may be specified using the `xtrafo` argument. If, for example, a ranking *both* `x1` and `x2` is required,

```
> ctree(y ~ x1 + x2, data = ls, xtrafo = function(data) trafo(data,
+ numeric_trafo = rank))
```

can be used. The *influence function* $h : \mathcal{Y} \times \mathcal{Y}^n \to \mathbb{R}^q$ depends on the responses $(\mathbf{Y}_1, \dots, \mathbf{Y}_n)$ in a permutation symmetric way. Section~4 explains how to choose g_j and h in different practical settings. A $p_j \times q$ matrix is converted into a $p_j q$ column vector by column-wise combination using the 'vec' operator. The influence function can be specified using the `ytrafo` argument.

The distribution of $\mathbf{T}_j(\mathcal{L}_n, \mathbf{w})$ under H_0^j depends on the joint distribution of $\mathbf{Y}$ and X_j, which is unknown under almost all practical circumstances. At least under the null hypothesis one can dispose of this dependency by fixing the covariates and conditioning on all possible permutations of the responses. This principle leads to test procedures known as *permutation tests*. The conditional expectation $\mu_j \in \mathbb{R}^{p_j q}$ and covariance $\Sigma_j \in \mathbb{R}^{p_j q \times p_j q}$ of $\mathbf{T}_j(\mathcal{L}_n, \mathbf{w})$ under H_0 given all permutations $\sigma \in S(\mathcal{L}_n, \mathbf{w})$ of the responses are derived by Strasser and Weber (1999):

$$\begin{aligned}
\mu_j &= \mathbb{E}(\mathbf{T}_j(\mathcal{L}_n, \mathbf{w}) | S(\mathcal{L}_n, \mathbf{w})) = \text{vec}\left(\left(\sum_{i=1}^n w_i g_j(X_{ji}) \right) \mathbb{E}(h | S(\mathcal{L}_n, \mathbf{w}))^\top \right), \\
\Sigma_j &= \mathbb{V}(\mathbf{T}_j(\mathcal{L}_n, \mathbf{w}) | S(\mathcal{L}_n, \mathbf{w})) \\
&= \frac{\mathbf{w}.}{\mathbf{w}. - 1} \mathbb{V}(h | S(\mathcal{L}_n, \mathbf{w})) \otimes \left(\sum_i w_i g_j(X_{ji}) \otimes w_i g_j(X_{ji})^\top \right) \qquad (2) \\
&- \frac{1}{\mathbf{w}. - 1} \mathbb{V}(h | S(\mathcal{L}_n, \mathbf{w})) \otimes \left(\sum_i w_i g_j(X_{ji}) \right) \otimes \left(\sum_i w_i g_j(X_{ji}) \right)^\top
\end{aligned}$$

where $\mathbf{w}. = \sum_{i=1}^n w_i$ denotes the sum of the case weights, $\otimes$ is the Kronecker product and the conditional expectation of the influence function is

$$\mathbb{E}(h | S(\mathcal{L}_n, \mathbf{w})) = \mathbf{w}.^{-1} \sum_i w_i h(\mathbf{Y}_i, (\mathbf{Y}_1, \dots, \mathbf{Y}_n)) \in \mathbb{R}^q$$

with corresponding $q \times q$ covariance matrix

$$\begin{aligned}
\mathbb{V}(h | S(\mathcal{L}_n, \mathbf{w})) = \mathbf{w}.^{-1} \sum_i w_i & \left(h(\mathbf{Y}_i, (\mathbf{Y}_1, \dots, \mathbf{Y}_n)) - \mathbb{E}(h | S(\mathcal{L}_n, \mathbf{w})) \right) \\
& \left(h(\mathbf{Y}_i, (\mathbf{Y}_1, \dots, \mathbf{Y}_n)) - \mathbb{E}(h | S(\mathcal{L}_n, \mathbf{w})) \right)^\top .
\end{aligned}$$

Having the conditional expectation and covariance at hand we are able to standardize a linear statistic $\mathbf{T} \in \mathbb{R}^{pq}$ of the form (1) for some $p \in \{p_1, \dots, p_m\}$. Univariate test statistics~c mapping an observed multivariate linear statistic $\mathbf{t} \in \mathbb{R}^{pq}$ into the real line can be of arbitrary form. An obvious choice is the maximum of the absolute values of the standardized linear statistic

$$c_{\max}(\mathbf{t}, \mu, \Sigma) = \max_{k=1,\dots,pq} \left| \frac{(\mathbf{t} - \mu)_k}{\sqrt{(\Sigma)_{kk}}} \right|$$

utilizing the conditional expectation μ and covariance matrix Σ. The application of a quadratic form $c_{\text{quad}}(\mathbf{t}, \mu, \Sigma) = (\mathbf{t} - \mu)\Sigma^+(\mathbf{t} - \mu)^\top$ is one alternative, although computationally more expensive because the Moore-Penrose inverse Σ^+ of Σ is involved.

The type of test statistic to be used can be specified by means of the `ctree_control` function, for example

```
R> ctree(y ~ x1 + x2, data = ls,
      control = ctree_control(teststat = "max"))
```

uses $c_{\max}$ and

```
R> ctree(y ~ x1 + x2, data = ls,
      control = ctree_control(teststat = "quad"))
```

takes c_{quad} (the default).

It is important to note that the test statistics $c(\mathbf{t}_j, \mu_j, \Sigma_j), j = 1, \dots, m$, cannot be directly compared in an unbiased way unless all of the covariates are measured at the same scale, i.e., $p_1 = p_j, j = 2, \dots, m$. In order to allow for an unbiased variable selection we need to switch to the P-value scale because P-values for the conditional distribution of test statistics $c(\mathbf{T}_j(\mathcal{L}_n, \mathbf{w}), \mu_j, \Sigma_j)$ can be directly compared among covariates measured at different scales. In step 1 of the generic algorithm we select the covariate with minimum P-value, i.e., the covariate X_{j^*} with $j^* = \text{argmin}_{j=1,\dots,m} P_j$, where

$$P_j = \mathbb{P}_{H_0^j}(c(\mathbf{T}_j(\mathcal{L}_n, \mathbf{w}), \mu_j, \Sigma_j) \geq c(\mathbf{t}_j, \mu_j, \Sigma_j) | S(\mathcal{L}_n, \mathbf{w}))$$

denotes the P-value of the conditional test for H_0^j. So far, we have only addressed testing each partial hypothesis H_0^j, which is sufficient for an unbiased variable selection. A global test for H_0 required in step 1 can be constructed via an aggregation of the transformations $g_j, j = 1, \dots, m$, i.e., using a linear statistic of the form

$$\mathbf{T}(\mathcal{L}_n, \mathbf{w}) = \text{vec}\left(\sum_{i=1}^{n} w_i \left(g_1(X_{1i})^\top, \dots, g_m(X_{mi})^\top\right)^\top h(\mathbf{Y}_i, (\mathbf{Y}_1, \dots, \mathbf{Y}_n))^\top\right).$$

However, this approach is less attractive for learning samples with missing values. Universally applicable approaches are multiple test procedures based on $P_1, \dots, P_m$. Simple Bonferroni-adjusted P-values (mP_j prior to version 0.8-4, now $1 - (1 - P_j)^m$ is used), available via

```
> ctree_control(testtype = "Bonferroni")
```

or a min-P-value resampling approach

```
> ctree_control(testtype = "MonteCarlo")
```

are just examples and we refer to the multiple testing literature (e.g., Westfall and Young 1993) for more advanced methods. We reject H_0 when the minimum of the adjusted P-values is less than a pre-specified nominal level α and otherwise stop the algorithm. In this sense, α may be seen as a unique parameter determining the size of the resulting trees.

3.2. Splitting criteria

Once we have selected a covariate in step 1 of the algorithm, the split itself can be established by any split criterion, including those established by Breiman *et˜al.* (1984) or Shih (1999). Instead of simple binary splits, multiway splits can be implemented as well, for example utilizing the work of O'Brien (2004). However, most splitting criteria are not applicable to

response variables measured at arbitrary scales and we therefore utilize the permutation test framework described above to find the optimal binary split in one selected covariate X_{j^*} in step~2 of the generic algorithm. The goodness of a split is evaluated by two-sample linear statistics which are special cases of the linear statistic (1). For all possible subsets A of the sample space $\mathcal{X}_{j^*}$ the linear statistic

$$\mathbf{T}_{j^*}^A(\mathcal{L}_n, \mathbf{w}) = \text{vec}\left(\sum_{i=1}^n w_i I(X_{j^*i} \in A) h(\mathbf{Y}_i, (\mathbf{Y}_1, \dots, \mathbf{Y}_n))^\top\right) \in \mathbb{R}^q$$

induces a two-sample statistic measuring the discrepancy between the samples $\{\mathbf{Y}_i | w_i > 0 \text{ and } X_{ji} \in A; i = 1, \dots, n\}$ and $\{\mathbf{Y}_i | w_i > 0 \text{ and } X_{ji} \not\in A; i = 1, \dots, n\}$. The conditional expectation $\mu_{j^*}^A$ and covariance $\Sigma_{j^*}^A$ can be computed by (2). The split A^* with a test statistic maximized over all possible subsets A is established:

$$A^* = \operatorname*{argmax}_A c(\mathbf{t}_{j^*}^A, \mu_{j^*}^A, \Sigma_{j^*}^A). \quad (3)$$

The statistics $c(\mathbf{t}_{j^*}^A, \mu_{j^*}^A, \Sigma_{j^*}^A)$ are available for each node with

```
> ctree_control(savesplitstats = TRUE)
```

and can be used to depict a scatter plot of the covariate $\mathcal{X}_{j^*}$ against the statistics.

Note that we do not need to compute the distribution of $c(\mathbf{t}_{j^*}^A, \mu_{j^*}^A, \Sigma_{j^*}^A)$ in step~2. In order to anticipate pathological splits one can restrict the number of possible subsets that are evaluated, for example by introducing restrictions on the sample size or the sum of the case weights in each of the two groups of observations induced by a possible split. For example,

```
> ctree_control(minsplit = 20)
```

requires the sum of the weights in both the left and right daughter node to exceed the value of 20.

3.3. Missing values and surrogate splits

If an observation X_{ji} in covariate X_j is missing, we set the corresponding case weight w_i to zero for the computation of $\mathbf{T}_j(\mathcal{L}_n, \mathbf{w})$ and, if we would like to split in X_j, in $\mathbf{T}_j^A(\mathcal{L}_n, \mathbf{w})$ as well. Once a split A^* in X_j has been implemented, surrogate splits can be established by searching for a split leading to roughly the same division of the observations as the original split. One simply replaces the original response variable by a binary variable $I(X_{ji} \in A^*)$ coding the split and proceeds as described in the previous part. The number of surrogate splits can be controlled using

```
> ctree_control(maxsurrogate = 3)
```

3.4. Inspecting a tree

Once we have fitted a conditional tree via

```
> ct <- ctree(y ~ x1 + x2, data = ls)
```

we can inspect the results via a `print` method

```
> ct

         Conditional inference tree with 2 terminal nodes

Response:  y
Inputs:  x1, x2
Number of observations:  150

1) x1 <= 0.8255248; criterion = 1, statistic = 22.991
  2)*  weights = 96
1) x1 > 0.8255248
  3)*  weights = 54
```

or by looking at a graphical representation as in Figure˜1.

```
> plot(ct)
```

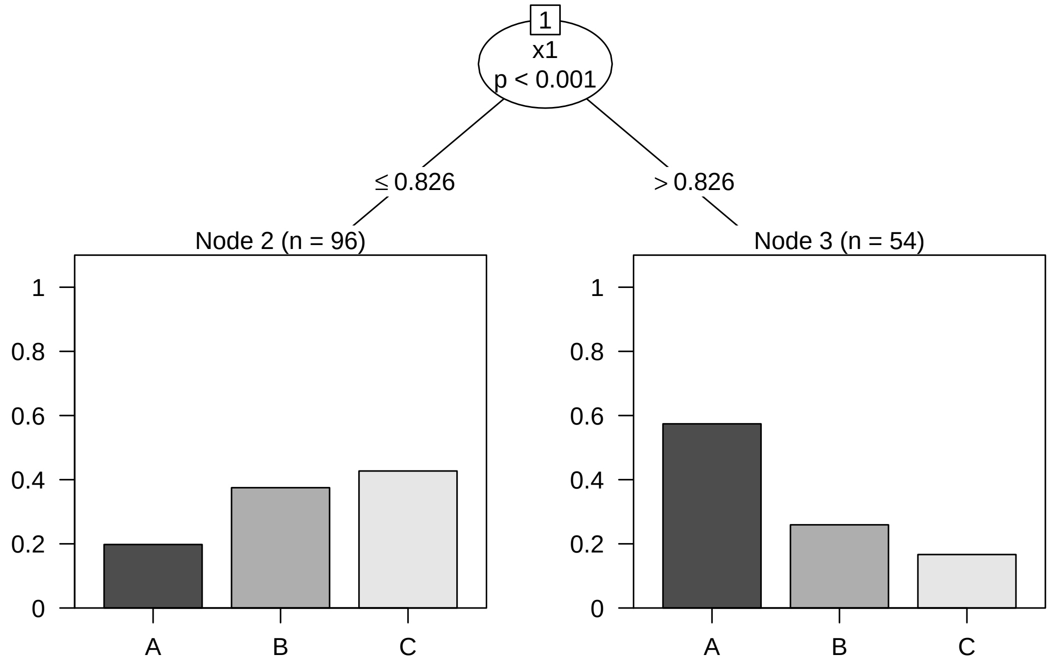

Figure 1: A graphical representation of a classification tree.

Each node can be extracted by its node number, i.e., the root node is

```
> nodes(ct, 1)
```

```
[[1]]
1) x1 <= 0.8255248; criterion = 1, statistic = 22.991
  2)*  weights = 96
1) x1 > 0.8255248
  3)*  weights = 54
```

This object is a conventional list with elements

```
> names(nodes(ct, 1)[[1]])
```

```
 [1] "nodeID"     "weights"    "criterion"  "terminal"   "psplit"
 [6] "ssplits"    "prediction" "left"       "right"      NA
```

and we refer to the manual pages for a description of those elements. The `Predict` function aims at computing predictions in the space of the response variable, in our case a factor

```
> Predict(ct, newdata = ls)
```

```
  [1] A A A A C A C A C C A A C A A A A C A C A A A C A A A C C A A C
 [33] A A C A A C C C A A C C C C A A A A A A C C C C A C C A C C C C
 [65] C C A A A A A C C A C A C C C C C C C C C C C A C A C A C C C
 [97] C C C C C A C C C A C C A C C C C C C C A C C C C C C C C C C C
[129] C C C C C C C C C A C C C C A C C A C A C A
Levels: A B C
```

When we are interested in properties of the conditional distribution of the response given the covariates, we use

```
> treeresponse(ct, newdata = ls[c(1, 51, 101),])
```

```
[[1]]
[1] 0.5740741 0.2592593 0.1666667

[[2]]
[1] 0.5740741 0.2592593 0.1666667

[[3]]
[1] 0.1979167 0.3750000 0.4270833
```

which, in our case, is a list with conditional class probabilities. We can determine the node numbers of nodes some new observations are falling into by

```
> where(ct, newdata = ls[c(1,51,101),])
```

```
[1] 3 3 2
```

4. Examples

4.1. Univariate continuous or discrete regression

For a univariate numeric response $\mathbf{Y} \in \mathbb{R}$, the most natural influence function is the identity $h(\mathbf{Y}_i, (\mathbf{Y}_1, \dots, \mathbf{Y}_n)) = \mathbf{Y}_i$. In case some observations with extremely large or small values have been observed, a ranking of the observations may be appropriate: $h(\mathbf{Y}_i, (\mathbf{Y}_1, \dots, \mathbf{Y}_n)) = \sum_{k=1}^{n} w_k I(\mathbf{Y}_k \leq \mathbf{Y}_i)$ for $i = 1, \dots, n$. Numeric covariates can be handled by the identity transformation $g_{ji}(x) = x$ (ranks are possible, too). Nominal covariates at levels $1, \dots, K$ are represented by $g_{ji}(k) = e_K(k)$, the unit vector of length K with kth element being equal to one. Due to this flexibility, special test procedures like the Spearman test, the Wilcoxon-Mann-Whitney test or the Kruskal-Wallis test and permutation tests based on ANOVA statistics or correlation coefficients are covered by this framework. Splits obtained from (3) maximize the absolute value of the standardized difference between two means of the values of the influence functions. For prediction, one is usually interested in an estimate of the expectation of the response $\mathbb{E}(\mathbf{Y}|\mathbf{X} = \mathbf{x})$ in each cell, an estimate can be obtained by

$$\hat{\mathbb{E}}(\mathbf{Y}|\mathbf{X} = \mathbf{x}) = \left(\sum_{i=1}^{n} w_i(\mathbf{x})\right)^{-1} \sum_{i=1}^{n} w_i(\mathbf{x})\mathbf{Y}_i.$$

4.2. Censored regression

The influence function h may be chosen as Logrank or Savage scores taking censoring into account and one can proceed as for univariate continuous regression. This is essentially the approach first published by Segal (1988). An alternative is the weighting scheme suggested by Molinaro, Dudoit, and van˜der Laan (2004). A weighted Kaplan-Meier curve for the case weights $\mathbf{w}(\mathbf{x})$ can serve as prediction.

4.3. J-class classification

The nominal response variable at levels $1, \dots, J$ is handled by influence functions $h(\mathbf{Y}_i, (\mathbf{Y}_1, \dots, \mathbf{Y}_n)) = e_J(\mathbf{Y}_i)$. Note that for a nominal covariate X_j at levels $1, \dots, K$ with $g_{ji}(k) = e_K(k)$ the corresponding linear statistic $\mathbf{T}_j$ is a vectorized contingency table. The conditional class probabilities can be estimated via

$$\hat{\mathbb{P}}(\mathbf{Y} = y|\mathbf{X} = \mathbf{x}) = \left(\sum_{i=1}^{n} w_i(\mathbf{x})\right)^{-1} \sum_{i=1}^{n} w_i(\mathbf{x}) I(\mathbf{Y}_i = y), \quad y = 1, \dots, J.$$

4.4. Ordinal regression

Ordinal response variables measured at J levels, and ordinal covariates measured at K levels, are associated with score vectors $\xi \in \mathbb{R}^J$ and $\gamma \in \mathbb{R}^K$, respectively. Those scores reflect the 'distances' between the levels: If the variable is derived from an underlying continuous variable, the scores can be chosen as the midpoints of the intervals defining the levels. The

linear statistic is now a linear combination of the linear statistic $\mathbf{T}_j$ of the form

$$\mathbf{MT}_j(\mathcal{L}_n, \mathbf{w}) = \text{vec}\left(\sum_{i=1}^{n} w_i \gamma^\top g_j(X_{ji}) \left(\xi^\top h(\mathbf{Y}_i, (\mathbf{Y}_1, \dots, \mathbf{Y}_n)\right)^\top\right)$$

with $g_j(x) = e_K(x)$ and $h(\mathbf{Y}_i, (\mathbf{Y}_1, \dots, \mathbf{Y}_n)) = e_J(\mathbf{Y}_i)$. If both response and covariate are ordinal, the matrix of coefficients is given by the Kronecker product of both score vectors $\mathbf{M} = \xi \otimes \gamma \in \mathbb{R}^{1,KJ}$. In case the response is ordinal only, the matrix of coefficients $\mathbf{M}$ is a block matrix

$$\mathbf{M} = \left(\begin{array}{ccc|c|ccc} \xi_1 & & 0 & & \xi_q & & 0 \\ & \ddots & & \dots & & \ddots & \\ 0 & & \xi_1 & & 0 & & \xi_q \end{array}\right) \text{ or } \mathbf{M} = \text{diag}(\gamma)$$

when one covariate is ordered but the response is not. For both $\mathbf{Y}$ and X_j being ordinal, the corresponding test is known as linear-by-linear association test (Agresti 2002). Scores can be supplied to `ctree` using the `scores` argument, see Section˜5 for an example.

4.5. Multivariate regression

For multivariate responses, the influence function is a combination of influence functions appropriate for any of the univariate response variables discussed in the previous paragraphs, e.g., indicators for multiple binary responses (Zhang 1998; Noh, Song, and Park 2004), Logrank or Savage scores for multiple failure times and the original observations or a rank transformation for multivariate regression (De'ath 2002).

5. Illustrations and applications

In this section, we present regression problems which illustrate the potential fields of application of the methodology. Conditional inference trees based on c_quad-type test statistics using the identity influence function for numeric responses and asymptotic χ^2 distribution are applied. For the stopping criterion a simple Bonferroni correction is used and we follow the usual convention by choosing the nominal level of the conditional independence tests as $\alpha = 0.05$.

5.1. Tree pipit abundance

```
> data("treepipit", package = "coin")
> tptree <- ctree(counts ~ ., data = treepipit)
```

The impact of certain environmental factors on the population density of the tree pipit *Anthus trivialis* is investigated by Müller and Hothorn (2004). The occurrence of tree pipits was recorded several times at $n = 86$ stands which were established on a long environmental gradient. Among nine environmental factors, the covariate showing the largest association to the number of tree pipits is the canopy overstorey (P =0.002). Two groups of stands can be distinguished: Sunny stands with less than 40% canopy overstorey ($n = 24$) show

```
> plot(tptree, terminal_panel = node_hist(tptree, breaks = 0:6-0.5, ymax = 65,
+ horizontal = FALSE, freq = TRUE))
```

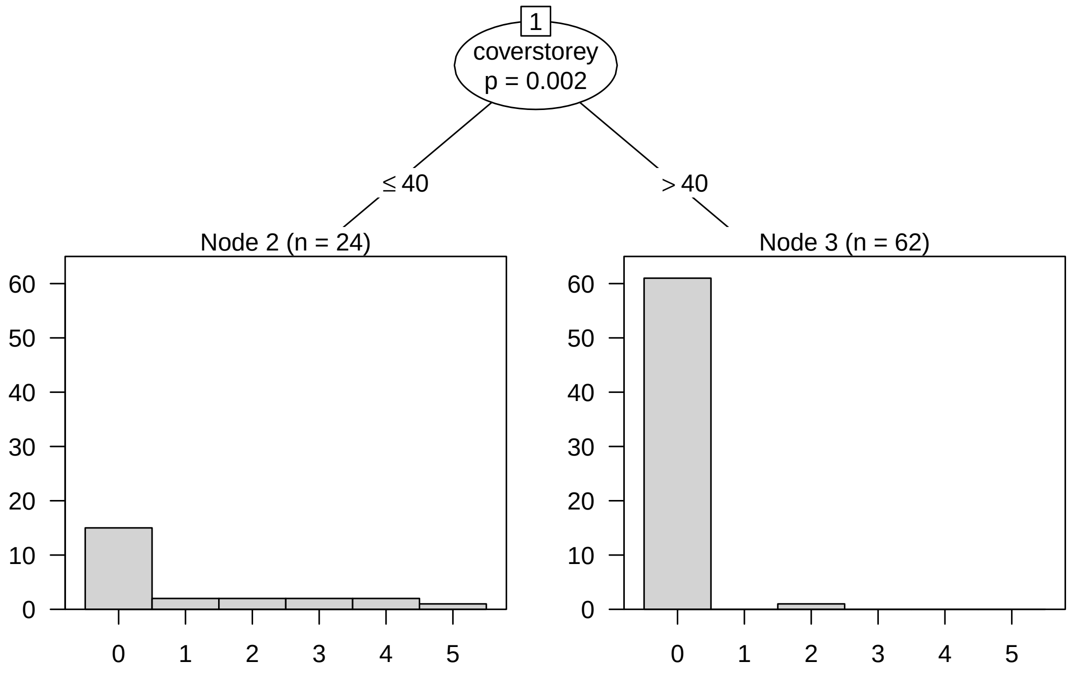

Figure 2: Conditional regression tree for the tree pipit data.

a significantly higher density of tree pipits compared to darker stands with more than 40% canopy overstorey ($n = 62$). This result is important for management decisions in forestry enterprises: Cutting the overstorey with release of old oaks creates a perfect habitat for this indicator species of near natural forest environments.

5.2. Glaucoma and laser scanning images

```
> data("GlaucomaM", package = "TH.data")
> gtree <- ctree(Class ~ ., data = GlaucomaM)
```

Laser scanning images taken from the eye background are expected to serve as the basis of an automated system for glaucoma diagnosis. Although prediction is more important in this application (Mardin, Hothorn, Peters, Jünemann, Nguyen, and Lausen 2003), a simple visualization of the regression relationship is useful for comparing the structures inherent in the learning sample with subject matter knowledge. For 98 patients and 98 controls, matched by age and gender, 62 covariates describing the eye morphology are available. The data is part of the **TH.data** package, `http://CRAN.R-project.org`). The first split in Figure~3 separates eyes with a volume above reference less than 0.059 mm^3 in the inferior part of the optic nerve head (`vari`). Observations with larger volume are mostly controls, a finding

which corresponds to subject matter knowledge: The volume above reference measures the thickness of the nerve layer, expected to decrease with a glaucomatous damage of the optic nerve. Further separation is achieved by the volume above surface global (`vasg`) and the volume above reference in the temporal part of the optic nerve head (`vart`).

The plot in Figure~3 is generated by

```
> plot(gtree)
```

and shows the distribution of the classes in the terminal nodes. This distribution can be shown for the inner nodes as well, namely by specifying the appropriate panel generating function (`node_barplot` in our case), see Figure~4.

```
> plot(gtree, inner_panel = node_barplot,
+      edge_panel = function(...) invisible(), tnex = 1)
```

As mentioned in Section~3, it might be interesting to have a look at the split statistics the split point estimate was derived from. Those statistics can be extracted from the `splitstatistic` element of a split and one can easily produce scatterplots against the selected covariate. For all three inner nodes of `gtree`, we produce such a plot in Figure~5. For the root node, the estimated split point seems very natural, since the process of split statistics seems to have a clear maximum indicating that the simple split point model is something reasonable to assume here. This is less obvious for nodes 2 and, especially, 3.

The class predictions of the tree for the learning sample (and for new observations as well) can be computed using the `Predict` function. A comparison with the true class memberships is done by

```
> table(Predict(gtree), GlaucomaM$Class)

           glaucoma normal
  glaucoma       74      5
  normal         24     93
```

When we are interested in conditional class probabilities, the `treeresponse` method must be used. A graphical representation is shown in Figure~6.

5.3. Node positive breast cancer

```
> data("GBSG2", package = "TH.data")
> stree <- ctree(Surv(time, cens) ~ ., data = GBSG2)
```

Recursive partitioning for censored responses has attracted a lot of interest (e.g., Segal 1988; LeBlanc and Crowley 1992). Survival trees using P-value adjusted Logrank statistics are used by Schumacher, Holländer, Schwarzer, and Sauerbrei (2001) for the evaluation of prognostic factors for the German Breast Cancer Study Group (GBSG2) data, a prospective controlled clinical trial on the treatment of node positive breast cancer patients. Here, we use Logrank

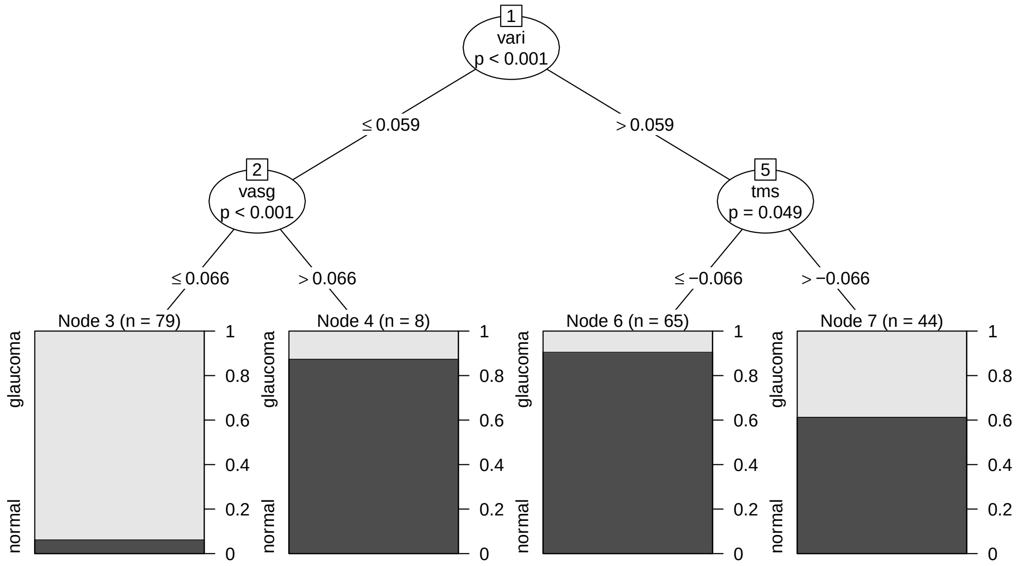

Figure 3: Conditional inference tree for the glaucoma data. For each inner node, the Bonferroni-adjusted P-values are given, the fraction of glaucomatous eyes is displayed for each terminal node. (Note: node 7 was not splitted prior to version 0.8-4 because of using another formulation of the Bonferroni-adjustment.)

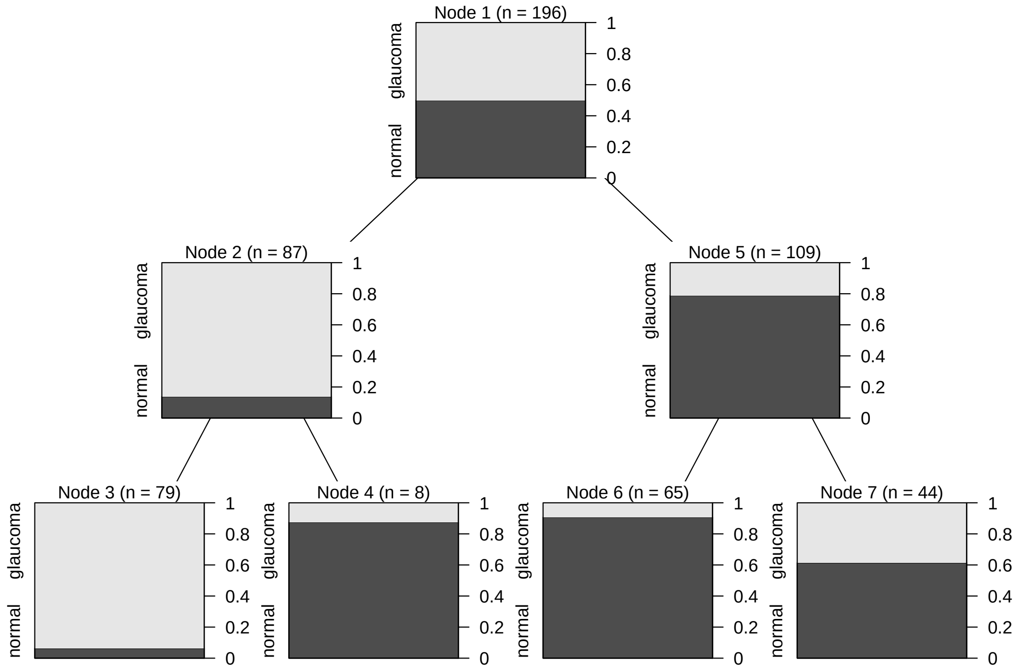

Figure 4: Conditional inference tree for the glaucoma data with the fraction of glaucomatous eyes displayed for both inner and terminal nodes.

```
> cex <- 1.6
> inner <- nodes(gtree, c(1, 2, 5))
> layout(matrix(1:length(inner), ncol = length(inner)))
> out <- sapply(inner, function(i) {
+     splitstat <- i$psplit$splitstatistic
+     x <- GlaucomaM[[i$psplit$variableName]][splitstat > 0]
+     plot(x, splitstat[splitstat > 0], main = paste("Node", i$nodeID),
+          xlab = i$psplit$variableName, ylab = "Statistic", ylim = c(0, 10),
+          cex.axis = cex, cex.lab = cex, cex.main = cex)
+     abline(v = i$psplit$splitpoint, lty = 3)
+ })
```

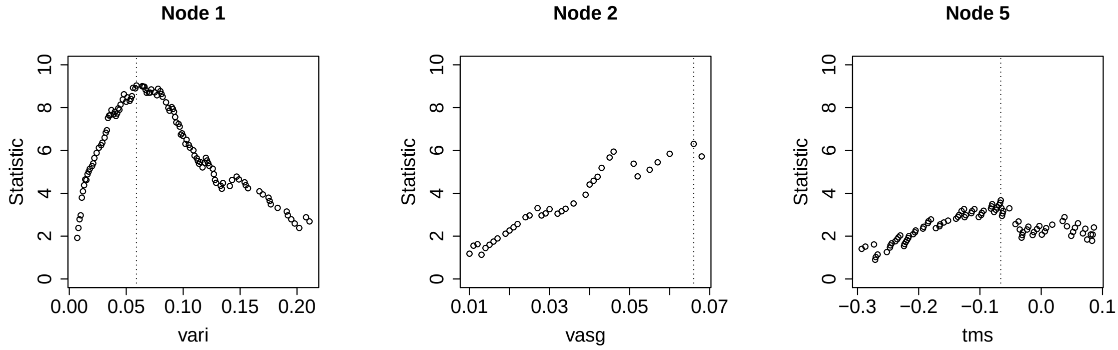

Figure 5: Split point estimation in each inner node. The process of the standardized two-sample test statistics for each possible split point in the selected input variable is show. The estimated split point is given as vertical dotted line.

scores as well. Complete data of seven prognostic factors of 686 women are used for prognostic modeling, the dataset is available within the **TH.data** package. The number of positive lymph nodes (`pnodes`) and the progesterone receptor (`progrec`) have been identified as prognostic factors in the survival tree analysis by Schumacher *et˜al.* (2001). Here, the binary variable coding whether a hormonal therapy was applied or not (`horTh`) additionally is part of the model depicted in Figure˜7.

The estimated median survival time for new patients is less informative compared to the whole Kaplan-Meier curve estimated from the patients in the learning sample for each terminal node. We can compute those 'predictions' by means of the `treeresponse` method

```
> treeresponse(stree, newdata = GBSG2[1:2,])

[[1]]
Call: survfit(formula = y ~ 1, weights = weights)

records   n.max n.start  events  median 0.95LCL 0.95UCL
    248     248     248      88    2093    1814      NA
```

```
> prob <- sapply(treeresponse(gtree), function(x) x[1]) +
+                  runif(nrow(GlaucomaM), min = -0.01, max = 0.01)
> splitvar <- nodes(gtree, 1)[[1]]$psplit$variableName
> plot(GlaucomaM[[splitvar]], prob,
+      pch = as.numeric(GlaucomaM$Class), ylab = "Conditional Class Prob.",
+      xlab = splitvar)
> abline(v = nodes(gtree, 1)[[1]]$psplit$splitpoint, lty = 2)
> legend(0.15, 0.7, pch = 1:2, legend = levels(GlaucomaM$Class), bty = "n")
```

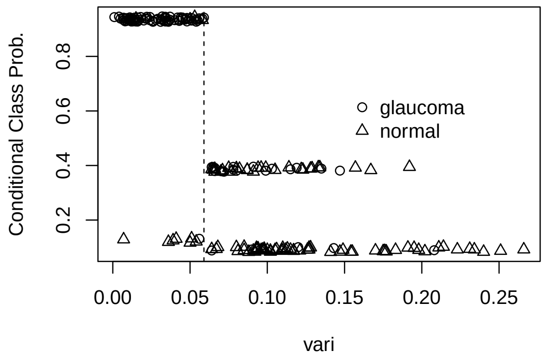

Figure 6: Estimated conditional class probabilities (slightly jittered) for the Glaucoma data depending on the first split variable. The vertical line denotes the first split point.

```
[[2]]
Call: survfit(formula = y ~ 1, weights = weights)

records   n.max n.start  events  median 0.95LCL 0.95UCL
    166     166     166      77    1701    1174    2018
```

5.4. Mammography experience

```
> mtree <- ctree(ME ~ ., data = mammoexp)
```

Ordinal response variables are common in investigations where the response is a subjective human interpretation. We use an example given by Hosmer and Lemeshow (2000), p. 264, studying the relationship between the mammography experience (never, within a year, over one year) and opinions about mammography expressed in questionnaires answered by $n = 412$ women. The resulting partition based on scores $\xi = (1, 2, 3)$ is given in Figure˜8. Women who (strongly) agree with the question ‘You do not need a mammogram unless you develop symptoms’ seldomly have experienced a mammography. The variable `benefit` is a score with

```
> plot(stree)
```

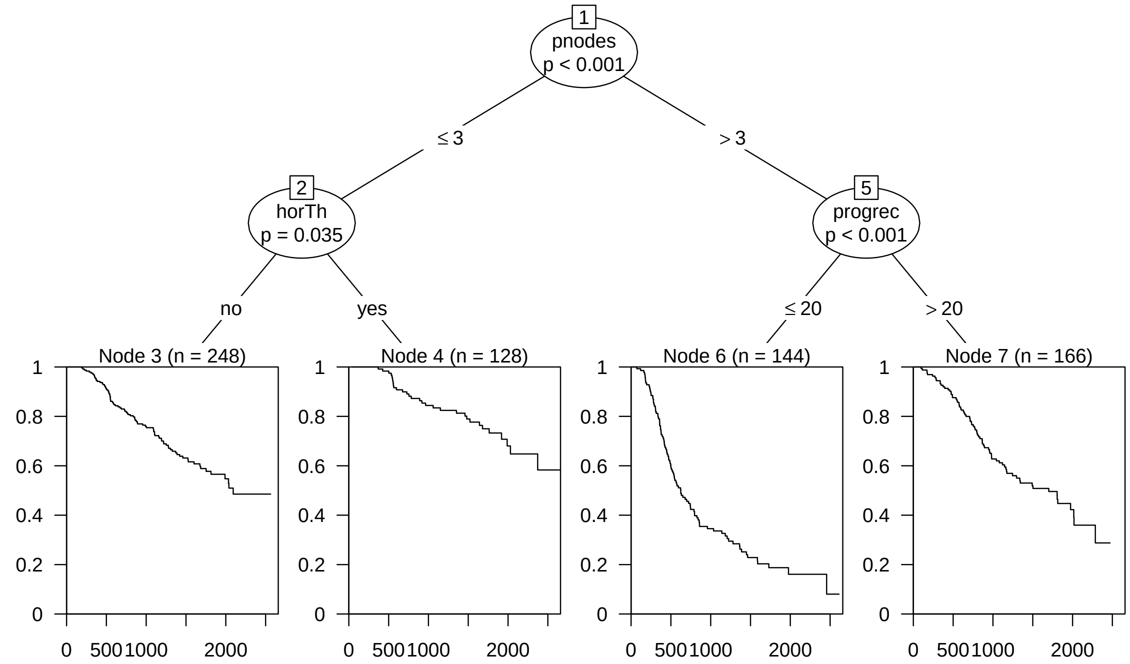

Figure 7: Tree-structured survival model for the GBSG2 data and the distribution of survival times in the terminal nodes. The median survival time is displayed in each terminal node of the tree.

low values indicating a strong agreement with the benefits of the examination. For those women in (strong) disagreement with the first question above, low values of `benefit` identify persons being more likely to have experienced such an examination at all.

References

Agresti A (2002). *Categorical Data Analysis*. 2nd edition. John Wiley & Sons, Hoboken, New Jersey.

Breiman L, Friedman JH, Olshen RA, Stone CJ (1984). *Classification and regression trees*. Wadsworth, California.

De'ath G (2002). "Multivariate Regression Trees: A New Technique For Modeling Species-Environment Relationships." *Ecology*, **83**(4), 1105–1117.

Hosmer DW, Lemeshow S (2000). *Applied Logistic Regression*. 2nd edition. John Wiley & Sons, New York.

Hothorn T, Hornik K, Zeileis A (2006). "Unbiased Recursive Partitioning: A Conditional

```
> plot(mtree)
```

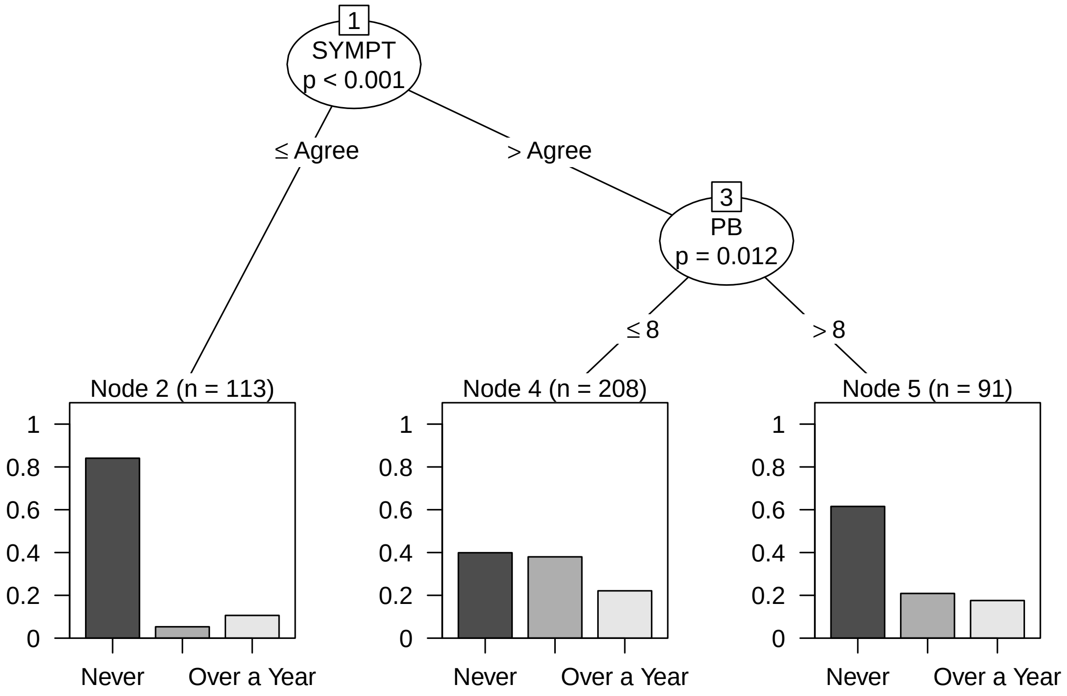

Figure 8: Ordinal regression for the mammography experience data with the fractions of (never, within a year, over one year) given in the nodes. No admissible split was found for node 4 because only 5 of 91 women reported a family history of breast cancer and the sample size restrictions would require more than 5 observations in each daughter node.

Inference Framework." *Journal of Computational and Graphical Statistics*, **15**(3), 651–674. doi:10.1198/106186006X133933.

LeBlanc M, Crowley J (1992). "Relative Risk Trees for Censored Survival Data." *Biometrics*, **48**, 411–425.

Mardin CY, Hothorn T, Peters A, Jünemann AG, Nguyen NX, Lausen B (2003). "New Glaucoma Classification Method based on standard HRT parameters by bagging classification trees." *Journal of Glaucoma*, **12**(4), 340–346.

Mingers J (1987). "Expert Systems – Rule Induction with Statistical Data." *Journal of the Operations Research Society*, **38**(1), 39–47.

Molinaro AM, Dudoit S, van˜der Laan MJ (2004). "Tree-Based Multivariate Regression and Density Estimation with Right-Censored Data." *Journal of Multivariate Analysis*, **90**(1), 154–177.

Müller J, Hothorn T (2004). "Maximally Selected Two-Sample Statistics as a new Tool for the Identification and Assessment of Habitat Factors with an Application to Breeding Bird Communities in Oak Forests." *European Journal of Forest Research*, **123**, 218–228.

Noh HG, Song MS, Park SH (2004). "An unbiased method for constructing multilabel classification trees." *Computational Statistics & Data Analysis*, **47**(1), 149–164.

O'Brien SM (2004). "Cutpoint Selection for Categorizing a Continuous Predictor." *Biometrics*, **60**, 504–509.

Quinlan JR (1993). *C4.5: Programs for Machine Learning*. Morgan Kaufmann Publ., San Mateo, California.

Schumacher M, Holländer N, Schwarzer G, Sauerbrei W (2001). "Prognostic Factor Studies." In J˜Crowley (ed.), *Statistics in Oncology*, pp. 321–378. Marcel Dekker, New York, Basel.

Segal MR (1988). "Regression Trees for Censored Data." *Biometrics*, **44**, 35–47.

Shih Y (1999). "Families of splitting criteria for classification trees." *Statistics and Computing*, **9**, 309–315.

Strasser H, Weber C (1999). "On the asymptotic theory of permutation statistics." *Mathematical Methods of Statistics*, **8**, 220–250.

Westfall PH, Young SS (1993). *Resampling-based Multiple Testing*. John Wiley & Sons, New York.

White AP, Liu WZ (1994). "Bias in Information-based Measures in Decision Tree Induction." *Machine Learning*, **15**, 321–329.

Zhang H (1998). "Classification Trees for Multiple Binary Responses." *Journal of the American Statistical Association*, **93**, 180–193.

Affiliation:

Torsten Hothorn
Institut für Statistik
Ludwig-Maximilians-Universität München
Ludwigstraße 33
80539 München, Germany
E-mail: `Torsten.Hothorn@R-project.org`
URL: `http://www.stat.uni-muenchen.de/~hothorn/`

Kurt Hornik, Achim Zeileis
Institute for Statistics and Mathematics
WU Wirtschaftsuniversität Wien
Augasse 2–6
1090 Wien, Austria
E-mail: `Kurt.Hornik@R-project.org`, `Achim.Zeileis@R-project.org`
URL: `http://statmath.wu.ac.at/~hornik/`,
`http://statmath.wu.ac.at/~zeileis/`
